# Modeling Wood Encroachment in Abandoned Grasslands in the Eifel National Park – Model Description and Testing

**DOI:** 10.1371/journal.pone.0113827

**Published:** 2014-12-10

**Authors:** Silvana Hudjetz, Gottfried Lennartz, Klara Krämer, Martina Roß-Nickoll, André Gergs, Thomas G. Preuss

**Affiliations:** 1 Institute for Environmental Research (Biology V), RWTH Aachen University, Worringerweg 1, 52074, Aachen, Germany; 2 Research Institute for Ecosystem Analysis and Assessment (gaiac) at the RWTH Aachen University, Kackertstr. 10, 52072, Aachen, Germany; DOE Pacific Northwest National Laboratory, United States of America

## Abstract

The degradation of natural and semi-natural landscapes has become a matter of global concern. In Germany, semi-natural grasslands belong to the most species-rich habitat types but have suffered heavily from changes in land use. After abandonment, the course of succession at a specific site is often difficult to predict because many processes interact. In order to support decision making when managing semi-natural grasslands in the Eifel National Park, we built the WoodS-Model (Woodland Succession Model). A multimodeling approach was used to integrate vegetation dynamics in both the herbaceous and shrub/tree layer. The cover of grasses and herbs was simulated in a compartment model, whereas bushes and trees were modelled in an individual-based manner. Both models worked and interacted in a spatially explicit, raster-based landscape. We present here the model description, parameterization and testing. We show highly detailed projections of the succession of a semi-natural grassland including the influence of initial vegetation composition, neighborhood interactions and ungulate browsing. We carefully weighted the single processes against each other and their relevance for landscape development under different scenarios, while explicitly considering specific site conditions. Model evaluation revealed that the model is able to emulate successional patterns as observed in the field as well as plausible results for different population densities of red deer. Important neighborhood interactions such as seed dispersal, the protection of seedlings from browsing ungulates by thorny bushes, and the inhibition of wood encroachment by the herbaceous layer, have been successfully reproduced. Therefore, not only a detailed model but also detailed initialization turned out to be important for spatially explicit projections of a given site. The advantage of the WoodS-Model is that it integrates these many mutually interacting processes of succession.

## Introduction

The degradation of natural and semi-natural landscapes has become a matter of global concern. This includes habitats in Western and Central Europe [1]–[3]. Semi-natural grasslands are amongst the most species-rich habitat types in Germany [4] but have suffered heavily from changes in land use [5]–[7]. For maintenance and restoration of species-rich grassland communities, adequate management is crucial; in many cases intensification or abandonment leads to a severe reduction in species diversity over the long term [4], [8]–[10]. In the case of abandonment, succession may lead to the development of persistent fallows formed by tall forbs or to a rapid encroachment of woodlands. Specific developments strongly depend on site conditions, initial states of vegetation and neighborhood interactions [11]–[15].

Due to the complex interactions of the many processes driving succession, it is difficult to predict wood encroachment at a specific site. Ecological modeling is considered a potentially powerful tool for ecological forecasting and its application, in the light of the increasing complexity and extent of the environmental problems [16]–[18]. Ecological models can provide an insight into how an ecosystem functions, and the interplay of the underlying key processes, which is essential for successful landscape management [19]. In recent decades, a lot of research has gone into the understanding and simulation of tree establishment and dynamics within closed forests, so that now whole families of forest succession models exist, some of which include the impact of ungulate browsing [20]–[25]. By contrast, models including wood-grassland dynamics have been restricted mainly to arid regions [26], [27]; to our knowledge, only a few models exist that simulate the encroachment of woods upon temperate grasslands in a process-based, spatially explicit way [28]–[30].

In order to assist in decision making when managing the vast semi-natural grassland areas in the Eifel National Park in Western Germany, we built the WoodS-Model as an extension of the previously described GraS-Model (Grassland Succession Model) [31]. The WoodS-Model simulates wood encroachment on grasslands on the landscape scale combining an individual-based (IBM) with a difference equation model. The purpose of this research was thus to develop a dynamic, spatially explicit landscape model, which integrates mutually interacting processes and can be used to predict vegetation development on a particular site. The objective of the current paper is to provide a detailed model description of the simulation of woody species and their interactions with herbaceous species. To simulate the development of the herbaceous species, we used the GraS-Model, which has previously been described and tested [31].

In order to test the model’s predictive capabilities, which is a prerequisite for use in decision making, simulation results are compared with observed vegetation data on the study site, demonstrating that the model is able to emulate successional patterns observed in the field. Additionally, we simulate the landscape development with different population densities of red deer (*Cervus elaphus* L.) and test whether the observed succession can be emulated with the current high browser density on site and whether simulations with lower densities produce plausible results, so that the model can be used to support decision making including wildlife management.

## Materials and Methods

### 2.1. Study site

The study site covers an area of about 1,500 ha on the Dreiborner Hochfläche plateau (50°33′ N, 6°23′ E). It rises from about 400 m above sea level in the NE to about 580 m in the SW. Mean annual temperatures and precipitation in the NE–SW area usually range between 7.8 and 6.8°C and 900 and 1100 mm, respectively. Predominant soils are acid, silty cambisols (pH_CaCl2_: 4–5) of shallow depth. The study site was kept unwooded by agricultural land use until 1946 and since then has been mown and grazed by sheep. In the northern and central part of the Dreiborner Hochfläche, extensive mowing and grazing have continued in order to conserve the grasslands. The southern part, by contrast, is no longer managed and is now open to natural succession [32]. The grassland areas of the Dreiborner Hochfläche are now dominated by mountainous hay meadows (*Geranio-Trisetetum*), mountain pastures (*Festuco-* and *Lolio-Cynosuretum*) and fallow grasslands dominated by tall grasses such as cocksfoot (*Dactylis glomerata* L.), common velvet (*Holcus lanatus* L.), red fescue *(Festuca rubra* L.) and tall oat (*Arrhenatherum elatius* L.) (vegetation mapping by the Eifel National Park [33] and our own surveys [34]). The establishment of common broom (*Cytisus scoparius* L.) was enhanced by the so-called “Schiffelwirtschaft”, a traditional type of agricultural land use in the Eifel, in which fields were alternately ploughed, grazed and frequently burned. Broom now dominates the scenery of the Dreiborner Hochfläche. Further shrubs include raspberry (*Rubus idaeus* L.), bramble (*Rubus* sp.), blackthorn (*Prunus spinosa* L.) and hawthorn (*Crataegus monogyna* J.). Forests of spruce (*Picea abies* L.), birch (*Betula pendula* R.), beech (*Fagus sylvatica* L.), oak (*Quercus robur* L.) and a few other woody species surround the plateau.

The model was established for semi-natural grasslands located in the center of the Eifel National Park, Germany. The permit for all fieldwork carried out for this study was issued by the Eifel National Park Administration (Landesbetrieb Wald und Holz NRW, Nationalparkforstamt Eifel). No plants or animals were disturbed and no plants or parts thereof were removed as the study only comprised non-invasive vegetation surveys.

### 2.2. Model description

In the following sections, the WoodS-Model is described based on the ODD (Overview, Design concepts and Details) protocol [35]. The source code was written using the Embarcadero Delphi IDE and compiler for Object Pascal, Borland Developer Studio 2009. All objects and processes described in the text correspond to objects and methods in the source code.

#### 2.2.1. Purpose

The main purpose of this model is to simulate the encroachment of woods on a grassland mosaic subject to various forms of land use as well as ungulate browsing in a dynamic, process-based manner. The model is intended as a decision support system for stakeholders dealing with the management of grasslands at a landscape level.

#### 2.2.2. Scales, entities and state variables

When dealing with a landscape model, different scales must be taken into account. Whereas bushes and trees can be distinguished as single individuals within the landscape, grasses and herbs are rather perceived as the sum of those plants. In the case of grasses, owing to clonal growth, individuals can be difficult to distinguish in the field. To account for the activity of these plants on different scales, the WoodS-Model has therefore been set up using a multimodeling approach of an IBM for woody species and a difference equation model for the herb layer, which is held to be a powerful approach in recent environmental modeling [36], [37].

**Figure 1:**
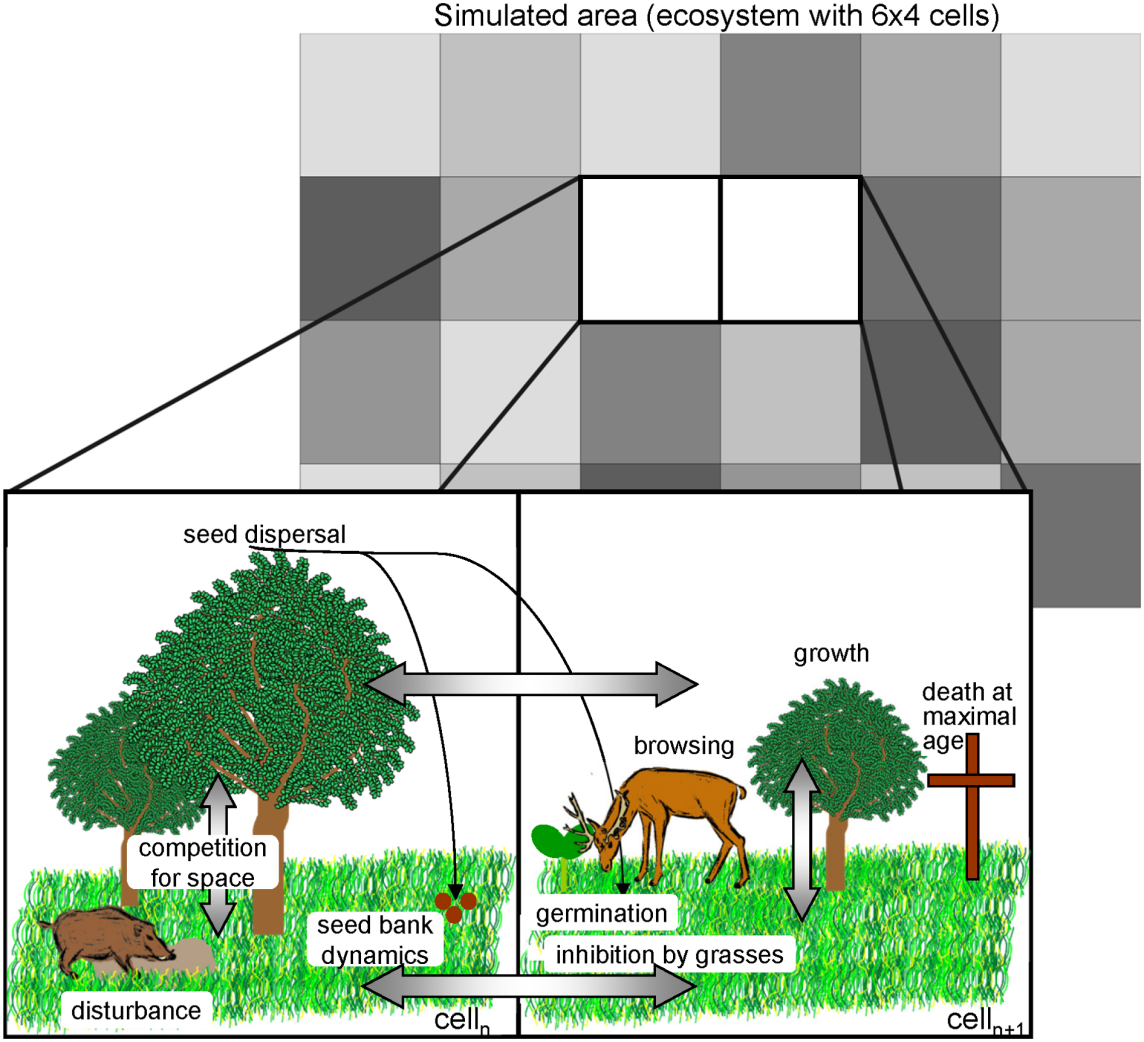
Multimodeling approach: Individually modelled bushes and trees are embedded in a grid-based difference equation model. All stated processes may take place in all cells. Arrows represent interactions.

Both herbaceous and woody species are embedded in a spatially explicit landscape grid (Fig. 1), which contains the spatio-temporal information within the model. We chose a fine grid with a cell size of 100 m^2^ to avoid artefacts that might occur when using a wider grid [38], e.g. when small but important features in the initial data such as field paths, hedges or small woods acting as propagule sources cannot be accounted for due to a large cell size, or when seed dispersal distances are shorter than cell length. Within this grid, the interactions between adjacent cells are managed, including seed dispersal and the vegetative spread of grasses and herbs [31]. Simulated landscapes can be large. The maximal size simulated so far was approximately 1,500 ha, i.e. 150,000 cells over a simulation time of 100 years calculated in daily time steps.

Each cell is characterized by its cell size, its X-/Y-coordinates, and the information about the form of land use (cutting, grazing, trampling), which are determined by the simulated scenario. In each cell, the growth of the herbaceous plant species and each individual agent is modelled. The agents of the WoodS-Model are the individual trees or bushes. They germinate, grow, suffer from browsing, produce and disperse seeds, and die when reaching their maximum age. Each bush and tree is characterized by species-specific parameters (Table 1). Each individual’s state variables are height, crown diameter, cover and age. For woody species, the composition of the herbaceous layer in each cell acts as an environmental variable (Table 1).

**Table 1:** Model components.

Model component	Type	Abbreviation	Unit
**Ecosystem**			
Number of cells	parameter		–
Simulated time	parameter		a
**Cells**			
Cell size	parameter	A	m^2^
X-Y-coordinates	parameter		–
Cutting intensity	disturbance variable	I_C_	–; ∈[0, 100]
Grazing intensity	disturbance variable	I_G_	–; ∈[0, 100]
Trampling intensity	disturbance variable	I_T_	–; ∈[0, 100]
Ungulate browsers	disturbance variable	UB	Ind 100 ha^−1^
Vegetation type	state variable		–
Seed bank	state variable		amount cell^−1^
**Bushes/trees**			
Growth rate	parameter	G	a^−1^
Maximal height	parameter	H_max_	m
Initial height	parameter	H_ini_	m
Maximal age	parameter	A_max_	a
Age of maturity	parameter	A_mat_	a
Allometric constant for crown diameter	parameter	A_crownconst_	–
Allometric coefficient for crown diameter	parameter	A_crowncoeff_	–
Allometric constant for biomass	parameter	A_biomconst_	–
Allometric coefficient for biomass	parameter	A_biomcoeff_	–
Effective seeding distance	parameter	ED	m
Maximum seeding distance	parameter	MD	m
Max. number of seeds	parameter	Seed_max_	–
Seed loss to predators	parameter	P_loss_	–
Germination rate	parameter	Germ	–
Decay time	parameter	Λ	a^−1^
Grass cover causing 50% inhibition	parameter	I50	m^2^
Slope of inhibition by grasses	parameter	S	–
Height	state variable	H	m
Crown diameter	state variable	D	m
Cover	state variable	C	m^2^
Age	state variable	A	a
Inhibition by grasses	state variable	I	–
**Grasses/herbs** (for model description see Siehoff et al. [31])			
Utilization indicator value for cutting	parameter	U_C_	–; ∈[1], [9]
Utilization indicator value for grazing	parameter	U_C_	–; ∈[1], [9]
Utilization indicator value for trampling	parameter	U_C_	–; ∈[1], [9]
Maximum growth rate	parameter	gmax	a^−1^
Factor for self-regulation	parameter	F_S_	–; ∈[0,10000]
Cover of each species	state variable	C	m^2^
Potential growth	state variable	(dc/dt)_pot_	m^2^ a^−1^
Growth to neighbour	state variable		m^2^ a^−1^
Realized growth	state variable	(dc/dt)_real_	m^2^ a^−1^

Species of the herbaceous layer are modelled at the population level using a difference equation approach. Their abundance is expressed as cover, so that single individuals are not distinguished and only vegetative spread of plants is considered. Model components of the herbaceous layer are provided in Table 1. The modeling approach of the herbaceous layer has been previously described in detail [31].

Shrub species under consideration in the WoodS-Model are broom, bramble and blackthorn, the most dominant species on the Dreiborner Hochfläche. As bramble and blackthorn distribute vegetatively via stolons, they are modelled in the same way as grasses and herbs. Dispersal via seeds has been omitted for these species. The parameters maximal growth rate (g_max_) and factor for self-regulation (F_s_), which determine the vegetative growth [31], were set to values at which the replacement of grasses and herbs in a fallow takes several decades which is typical for poor and temporarily dry sites (Table 2).

**Table 2:** Characteristics of bramble (*Rubus* sp.) and blackthorn (*Prunus spinosa*).

Species	U_C_ [–]	U_G_ [–]	U_T_ [–]	g_max_ [d^−1^]	F_S_ [–]
*Rubus sp.*	2	3	3	4.7	15000
*Prunus spinosa*	3	5	9	4.7	15000

U_C/G/T_: Utilization number for cutting, grazing or trampling; g_max_: maximum growth rate, F_S_: factor for self-regulation (for explanation see Siehoff et al. [31]).

#### 2.2.3. Process overview and scheduling

In the model, we considered processes of seed dispersal, seed decay, germination, growth and self-thinning in woody species. These processes depend on neighborhood interactions (seed dispersal determined by the landscape pattern) and on the given management regime (influencing the herbaceous layer and the density of ungulate browsers). Management is most important for grasses and herbs; only mowing has an influence on bushes and trees directly, as individuals between the size of 0.1 and 1 m die when the area is mown. Further environmental factors affecting the vegetation are disturbance by wild boar (*Sus scrofa* L.) and ungulate browsing.

**Figure 2:**
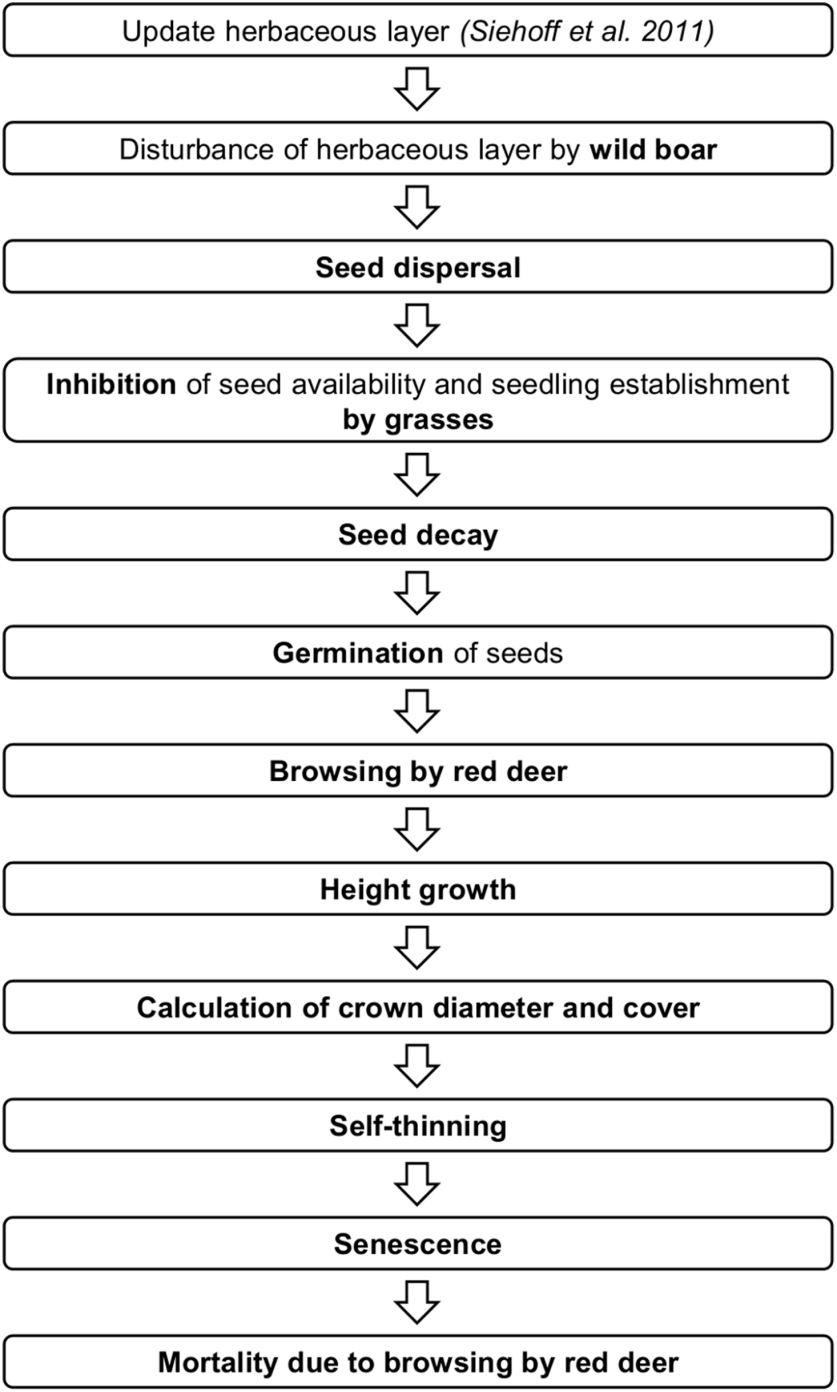
Flow chart of processes taking place in the individual-based WoodS-Model.

Landscape development is updated yearly, while the balance between processes is iterated in daily increments; however, no seasonal variability has been taken into account, as the model is not driven by any daily meteorological data. All cells are recalculated each increment from the upper left to the lower right. The processes are scheduled as shown in Fig. 2.

#### 2.2.4. Design concepts

*Basic principles*. Woody species are modelled in an individual-based manner [39], i.e. each individual is simulated based on its life cycle and its competition with other individuals for space. The herbaceous layer is implemented on the population scale using the GraS-Model [31]. The two models, the WoodS and the GraS, are embedded in a spatially explicit, raster-based environment (Fig. 1). The herbaceous layer mechanistically interacts with individual trees by decreasing the seed availability. In the model, browsing impacts on trees are explicitly modelled in terms of removed biomass and browsing-induced mortality. Ungulate browsing intensity is accounted for by comparing the supply and demand of woody browse. As a result, wood encroachment depends on the density of ungulates and on the potential abundance of sprouting young trees. The raster-based approach allows the insertion of spatially explicit input data of the actual landscape integrating initial vegetation composition and resulting neighborhood interactions, which are crucial for the course of succession of a given landscape [11]–[15]. Moreover, the approach allows the simulation of various management regimes to distinct areas of the modelled landscape. The model output can be linked with a GIS to visualized detailed raster maps, which is a precondition for the application by stakeholders who want to apply the model to existing landscapes [40].

*Emergence.* Bush and tree encroachment emerges from seed dispersal, seed decay, germination, growth, self-thinning, ungulate browsing and interactions with the herbaceous layer.

*Sensing.* In the self-thinning submodel, trees sense whether the resource space is limiting or not. When space is limiting, the smallest trees react by dying off. Browsing is sensed in the way that browsed trees cease to grow and may die at the end of the year. Furthermore, trees react to the composition of the herbaceous layer that hinders seeds from reaching the seed bank. The only management regime directly sensed by the trees is mowing, which kills saplings.

*Interaction.* Trees interact with their environment in terms of competition for space and the inhibition of germination by the herbaceous layer. Space is the resource that the individual trees in the WoodS-Model compete for. It is hereby used as a proxy to summarize different environmental resources such as light, nutrients and water. When space becomes limiting, self-thinning takes place and the smallest trees in one cell die. Trees are superior in succession over the bush common broom, which is modelled in the same way as the trees. Broom growth is restricted by bramble and blackthorn. Therefore, trees only compete with other tree individuals, whereas broom individuals compete with trees, bramble, blackthorn and conspecifics. Furthermore, seeds only germinate when there is space available in the given cell. For trees, available space is calculated as the space free from all trees (crown area) not including the bushes broom, bramble and blackthorn. For broom, available space only consists of the space free from trees and bushes. Competition for space only takes place within a cell, not between adjacent cells.

Tree recruitment can be strongly inhibited by the herbaceous layer, because it prevents the seeds from reaching the seed bank. Once a tree or bush has germinated, it is not hindered by grasses and herbs. Instead, it replaces grasses by virtue of the fact that space covered by trees and bushes is not available to grasses. Therefore, only the space free of trees and bushes is considered in all calculations concerning the herbaceous layer. In addition, trees are influenced by ungulate browsing.

*Stochasticity.* In order to take natural variation into account, several processes are calculated stochastically. When input data of a landscape is imported from a GIS map, scattered young trees and bushes are randomly clumped in grassland or shrubbery vegetation types. Once a year, some of the non-forest cells are disturbed by wild boar; disturbed cells are chosen randomly. Furthermore, the parameters of the trees’ life cycle are sampled from random distributions as follows: for each time step, the growth rate of each tree is randomly drawn from a normal distribution around its mean. For the calculation of number of seeds per tree, each year is randomly chosen to be a mast year with 70–100% of maximum amount of seeds, a year with 40–70% of seeds, a year with 10–40% of seeds or even a year without any seeds. The percentage of seeds is then randomly selected from the given range. The maximum age of a tree varies by +/−10% of the species’ maximum age for each individual. The mortality of trees due to browsing is implemented as a stochastic event.

#### 2.2.5. Initialization

For model testing, we use simplified synthetic landscapes, for which input data can be written into ASCII files. For a realistic scenario, the simulation was initialized with data of landscape and form of land use from a detailed vegetation map of the study site [33] (originally a polygon map that was converted into a raster map in a GIS as described below). For the initialization of the vegetation composition, data for the coverage of each modelled species is needed (for details for the herb layer see Siehoff et al. [34]). Therefore, a detailed vegetation mapping of the Dreiborner Hochfläche was carried out by the national park administration [33]. Areas with a tree cover over 50% were imported as forest cells. For grassland or bush areas with scattered young trees or bushes, the amount (percentage cover) of young trees and bushes was classified by age (trees: 1, 5 years; broom: 2, 7, 10 years). Trees were assumed to show a clumped distribution. Therefore, the percentage of cells in that polygon was randomly indicated as completely covered by trees of a given age, whereas the other cells contained no trees. Most groups of bushes on the study site did not completely cover an area of 100 m^2^( = 1 cell). To account for this observation, a proportion of cells equivalent to four times the percentage cover of bushes was assigned 25% cover of bushes for each polygon containing bushes, whilst the remainder of the cells in the polygon received zero cover.

#### 2.2.6. Observation

The output of the model contains information about numbers, heights and covers of all trees or bushes in each cell. The cover of the species is used to define the vegetation type of each cell. Vegetation dynamics can be plotted as cover of species in each cell and total cover of species in the whole area over time. Alternatively, vegetation types for given time steps can be used to illustrate simulation results in detailed raster maps using ArcGIS9 (ESRI).

### 2.3. Description of submodels

#### 2.3.1. Submodels for woody species

The simulation of woody species is divided into several submodels in accordance with their life cycle (Fig. 2). Seeds are dispersed and reach the seed bank when not prevented from doing so by the herb layer. Afterwards, seeds germinate and seedlings and young saplings are subject to browsing. Should this browsing not take place, they grow and might die due to subsequent browsing, competition for space (self-thinning) or senescence. If they reach the age of maturity, bushes and trees disperse seeds. The corresponding submodels and equations are described in the following section. Some of the subroutines were obtained from two forest models, TreeMig and LandClim. TreeMig is based on the well-tested model ForClim [41] and was itself tested for several scenarios using different temporal and spatial scales [42], [43]. LandClim is based on the well-established model LANDIS [44], [45] and has been tested for different regions [46]–[48]. We did not model all woody species existing on the Dreiborner Hochfläche, but focus on the most abundant species, namely birch, broom, beech, spruce and oak, as representatives.

*Seed dispersal.* Once a year, mature bushes and trees disperse their seed. For the calculation of number of seeds, each year is randomly chosen as a mast year with 70–100% of maximum amount of seeds, a year with 40–70% of seeds, a year with 10–40% of seeds or even a year without any seeds. The percentage of seeds is then randomly selected from the given range. Seeds are dispersed to the surrounding cells following the LANDIS-II double exponential seed dispersal algorithm [49], so that the dispersal kernel is characterized by the effective seeding distance (ED) and the maximum seeding distance (MD) [50]. The effective seeding distance is the farthest distance to which the majority (e.g. 95% for birch) of the seeds are dispersed, whereas the remaining seeds are distributed within the maximum distance [49]. The function is normalized so that its sum over all sink cells is one:



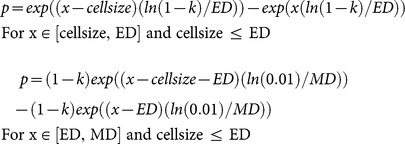



*p*: proportion of seeds landing in the sink cell

*x*: centroid-to-centroid distance from the source cell to the sink cell

cellsize: grid cell size (e.g. 10 m)

ED: effective distance

MD: maximum distance

k: probability that the seed will disperse within the effective distance (e.g. 0.95)

For broom, oak and beech, the maximum distance reflects zoochorous dispersal by mammals (e.g. red deer) and by birds (e.g. Eurasian jay *Garrulus glandarius* L.). As the jay prefers to store its seeds in small diverse woodland patches [51], zoochorously distributed acorns and beechnuts only land in a sink cell when this specific cell contains a minimum of one bush or tree, but is not a forest cell. Data for seed dispersal was derived from the literature as given in Table 3.

**Table 3:** Parameter values for the modelled tree and bush species.

Parameter	Abbreviation	Unit	Birch	Broom	Beech	Spruce	Oak
Growth rate	G (x ± s)	[a^−1^]	0.03±0.007 ^f)^	0.3±0.04 ^f)^	0.024±0.002	0.021±0.002 ^f)^	0.028±0.004 ^f)^
Maximal height	H_max_	[m]	30 ^1)^	3.7 ^2)^	45 ^1)^	45 ^1)^	35 ^1^*^)^
Initial height	H_ini_	[m]	0.05 ^f)^	0.05 ^f)^	0.05 ^f)^	0.05 ^f)^	0.05 ^f)^
Maximal age	A_max_	[a]	100 ^1)^	12 ^2)^	350 ^1)^	500 ^1)^	800 ^1)^
Age of maturity	A_mat_	[a]	15 ^3)^	4 ^4)^	50 ^3)^	45 ^3)^	50 ^3)^
Allometric constant forcrown diameter	A_cd_const_	[–]	1.146 ^f)^	0.945 ^f)^	0.716 ^f)^	0.880 ^f)^	1.144 ^f)^
Allometric coefficient forcrown diameter	A_cd_coeff_	[–]	0.791 ^f)^	1.236 ^f)^	1.073 ^f)^	0.877 ^f)^	0.929 ^f)^
Allometric constant forbiomass	A_biomconst_	[–]	0.0044 ^f)^	-	0.0250 ^f)^	0.1292 ^f)^	0.8172 ^f)^
Allometric coefficient forbiomass	A_biomcoeff_	[–]	1.247 ^f)^	-	2.180 ^f)^	2.569 ^f)^	3.746 ^f)^
Effective seeding distance	ED	[m]	200 ^8)^	10 ^14)^	30 ^8)^	70 ^8)^	30 ^8)^
Maximum seeding distance	MD	[m]	700 ^8)^	500 ^g,z)^	200 ^9,z)^	250 ^8)^	200 ^9,z)^
Proportion of seeds in ED	p_ED_	[–]	95 ^8)^	97 ^c,z)^	99 ^5,z)^	95 ^8)^	99 ^5,z)^
Max. number of seeds	Seed_max_	[–]	11,775,000 ^5)^	10000 ^4)^	29000 ^5)^	96500 ^5)^	27500 ^5)^
Seed loss to predators	P_loss_	[–]	0.8 ^5)^	0.8 ^5)^	0.8 ^5)^	0.8 ^5)^	0.8 ^ = 5)^
Germination rate	Germ	[–]	0.1 ^5)^	0.8 ^7)^	0.7 ^5)^	0.75 ^5)^	0.75 ^5)^
Decay time	λ	[a^−1^]	0.36 ^5)^	0.063 ^6)^	0.6 ^5)^	0.46 ^5)^	0.81 ^5)^
Browsing mortality	Brow_mort_	[–]	0.1 ^12, 13)^	-	0.2 ^10, 11)^	0.1 ^10, 12)^	0.2 ^11, 12)^
Grass cover causing50% inhibition	I_50_	[m^2^]	29.0^ c)^	26.1 ^c)^	29.5 ^c)^	29.3 ^c)^	29.8 ^c)^
Slope of inhibition by grasses	S	[–]	−10 ^c)^	−10 ^c)^	−10 ^c)^	−10 ^c)^	−10 ^c)^

x: mean, s: standard deviation.

References: ^1)^ [120], ^2)^ [67], ^3)^ [121], ^4)^ [122], ^5)^ [42], ^6)^ [104], ^7)^ [123], ^8)^ [46], ^9)^ [124], ^10)^ [95], ^11)^ [96], ^12)^ [125], ^13)^ [126], ^14)^ [127], [128]; *the source value was adjusted (not exceeding 15% deviation from the original value), ^f)^fit to own data, ^c)^manually calibrated (see text), ^g)^guess; ^z)^zoochorous.

*Inhibition by grasses.* Grasses (especially in fallows) may build up a thick layer of litter that prevents seeds from reaching the soil and hinders the germination and establishment of seedlings [11], [52]–[56]. For modeling the inhibition by grasses, we calculate an inhibition factor as a sigmoid function of grass cover, which is a proxy of different processes preventing seedling establishment by the herbaceous layer (see 4.1.3). The factors calculated for each single grass species, i.e. red fescue, tall oat, crested dog’s-tail (*Cynosurus cristatus* L.), perennial rye (*Lolium perenne* L.), cocksfoot and common velvet and the tufted plants (a group of perennial plants modelled in the herb layer [31]), are summed to total inhibition. This inhibition factor I (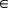
[0, 1]) is multiplied by the number of seeds reaching the seed bank.



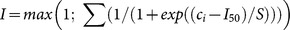



*I*: inhibition factor [–]

*n*: number of grasses

*c_i_*: cover of the grass species i [m^2^]

*I_50_*: value of grass cover that causes an inhibition of 50% [m^2^]

*S*: parameter defining the slope of the curve [–]

A thick grass felt develops mainly in fallow grasslands [54]. When the cell is mown or grazed, biomass is removed so that the grass sward is much weaker [57]. Therefore total inhibition is reduced by 30% for mown or grazed grasslands.

Before the seeds reach the seed bank in the sink cell, a high proportion (80%) of seeds are lost, due to non-specific predators such as mice [42]. Furthermore, grasses may hinder the seeds, so that the number of germinable seeds reaching the seed bank in one cell is calculated as:



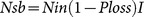



Nsb: number of seeds reaching the seed bank [–]

Nin: seeds incoming into the sink cell [–]

Ploss: proportion of seeds lost to unspecific predators [–]

I: inhibition by grasses [–]; 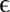 [0, 1]

The parameters I_50_ and S, which determine the inhibition of grasses for trees and broom, and the proportion of seeds in the effective seeding distance p(ED) for broom were visually calibrated, so that the simulation results met patterns of bush and tree encroachment observed on the Dreiborner Hochfläche. Data used for this calibration include field surveys and analyses of aerial photographs of various authors [57]–[62] and our own data collected for this study [34].

*Seed decay.* Following the TreeMig model [42], the decay of seeds in the seed bank is calculated exponentially with species-specific parameterization (Table 3):



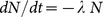



dN/dt: number of seeds decaying per time step

λ: decay constant [a^−1^]

N: number of seeds

*Germination.* Once a year, a fraction of the seeds in the seed bank germinates with species-specific parameterization (Table 3):



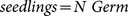



seedlings: number of seedlings [–]

N: number of seeds in the seed bank [–]

Germ: germination rate [–]

Seeds only germinate when there is available space in the given cell. For trees, available space is calculated as the space free of all trees, not including the bushes broom, bramble and blackthorn.



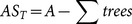



AS_T_: available space for trees [m^2^]

A: cell size [m^2^]

Σtrees: sum of cover of all trees [m^2^]

For broom, available space consists only of the space free of trees and all bushes:



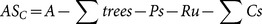



AS_C_: available space for broom C. scoparius [m^2^]

A: cell size [m^2^]

Σtrees: sum of cover of all trees [m^2^]

Ps: cover of blackthorn P. spinosa [m^2^]

Ru: cover of bramble Rubus sp. [m^2^]

ΣCs: sum of cover of all Cytisus scoparius bushes [m^2^]

The number of seeds germinating has been restricted to 35 tree plus 35 broom seedlings per cell to save simulation time and memory. The surplus of germinating seedlings is stored and added into the browsing equation. In fact, this artificial restriction anticipates actual browsing or self-thinning in the field. Germination rates for trees were taken from the TreeMig model [42]. The germination rate of birch was found to be too high. The high number of germinating birch seeds is strongly reduced in TreeMig by a high density-dependent mortality rate. This has not been implemented in the WoodS-Model. Therefore, the germination rate for birch was halved, resulting in a plausible seedling number for our study site.

*Height growth, crown diameter and cover.* Growth is modelled following a sigmoid growth curve, using a modified version of the Bertalanffy equation [63], [64]:



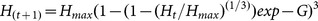



*H_(t+1)_*: height growth [m a^−1^]

*H_max_*: maximal height [m]

*G*: growth rate [a^−1^]

Mean values for the growth rate (G) of woody species were derived by fitting the height growth equation to yield tables [65] taking mean height values of the yield classes of the Eifel National Park into account (Vollmer pers. comm.). Data for young spruce (<10 a) was obtained from Nothdurft [66]. For broom, no raw data was available, but the equation was fitted to a regression model for data gathered from a similar climatic region in France [67].

In order to take natural variation into account, values for the model parameter G were sampled from a normal distribution that requires two parameters, the mean and the standard deviation. These parameters of the random distribution function were fitted to mean height values of the yield classes and to the edges of the yield classes, respectively. For broom, the approximate minimum and maximum values as provided by Prevosto et al. [67] were taken as two standard deviations.

Crown diameter is calculated as an allometric function of height [68]:



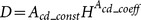



D: crown diameter [m]

H: height [m]

A_cd_const_: allometric constant to calculate crown diameter [–]

A_cd_coeff_: allometric coefficient to calculate crown diameter [–]

Based on crown diameter, the cover of the bush or tree is calculated as a circle:



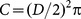



*C*: cover [m^2^]

Parameter values for the allometric constant (Aconst) and allometric coefficient (Acoeff) were derived by fitting the equation for the calculation of crown diameter to data of solitary bushes and trees in the Eifel National Park [69] and own measurements [34]; raw data is publicly available online.

*Self-thinning.* Space is the resource that the individual bushes and trees in the WoodS-Model compete for. Space is hereby used as a proxy to summarize different environmental resources such as light, nutrients and water. When space becomes limited, competition between bushes and trees results in self-thinning. Self-thinning is simulated according to Rammig et al. [64]: at the end of each time step (i.e. after growth), the crown areas of all bushes and trees in one cell are added together, and if the sum of crown areas exceeds the cell area, the smallest bush or tree dies. Common broom is inferior to trees during succession. Therefore, self-thinning is first calculated for broom, and subsequently for other woody species. Furthermore, broom also competes with bramble, blackthorn, other broom and with all tree individuals (i.e. the sum of the crown area is calculated over all broom and tree individuals plus the cover of bramble and blackthorn), whereas trees only compete with other trees. The competition between tree seedlings and bushes has not been taken into consideration, because at our site, protection from browsing with the current high population density of red deer (22 individuals per 100 ha) is likely to have a greater impact on the course of succession. This competition calculation is a simplification that we use because we do not model the vertical dimension of height and therefore light reduction for species in lower layers.

*Senescence.* Once a bush or tree reaches its maximal age (A_max_), it dies, i.e. the object is deleted. The maximum age varies by +/−10% of the species’ maximum age for each individual.

#### 2.3.2. Wild game submodels

*Disturbance by wild boar.* Wild boar (*Sus scrofa* L.) plough the ground when searching for food, creating open spots and facilitating wood encroachment. In the model, 10% of hay meadows and pastures, 3% of shrubs and 1% of fallow grassland cells are randomly chosen once a year, in which 45% of the herbaceous layer is removed (percentages as observed in the field [59]). As a consequence, the grass cover is reduced, leading to a lower inhibition by grasses (see above).

*Browsing by red deer.* Based on a food chain approach, we link the influence of ungulates on tree establishment with the actual animal density in a given area, similar to the FORSPACE model [70]. The impact of red deer (*C. elaphus*) on succession is simulated weighting the supply of available woody browse against the ungulate demand depending on ungulate density in each cell, with “supply” referred to as the parts of woody plants that are subject to browsing and “demand” referred to as the average annual woody part of the animals’ diet. Browsing impacts on trees are modelled in terms of removed biomass and browsing-induced mortality. As a result, wood encroachment depends on the density of ungulates and the potential abundance of sprouting young trees. In order to calculate the demand, first the weight of a “standard deer” is calculated as the mean weight of an individual in a “standard herd” consisting of five adult males (105 kg each), 10 adult females (70 kg each) and 20 juveniles (42.5 kg each) (average weights provided by Schulte) [71]. Demand of woody browse (in dry weight, dw) per individual is furthermore calculated as:



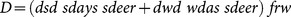



D: demand of woody browse [kg dw a^−1^]

dsd: daily summer demand [kg dw (100 kg red deer)^−1^]

sdays: number of summer days [–]

sdeer: weight of a “standard deer” [kg]

dwd: daily winter demand [kg dw (100 kg red deer)^−1^]

wdays: number of winter days [–]

frw: fraction of woody parts of the total food [–]

Data for calculating the demand for woody browse by red deer was gathered from the literature (Table 4). Data for open areas were chosen for parameterization [72]. When these values are substituted in the equation, it results in a demand for woody browse of 157.46 kg dw a^−1^ per “standard deer”. In simulations, we applied population densities of 0, 5, 10 and 22 red deer per 100 ha^−1^.

**Table 4:** Parameter values for calculating red deer demand for woody browse.

Parameter	Abbreviation	Unit	Value	Source
Daily summer demand	Dsd	[kg dw per 100 kg red deer]	2.5	[129]
Number of summer days	sdays	[–]	214	[61]
Daily winter demand	Dwd	[kg dw per 100 kg red deer]	3.0	[129]
Number of winter days	wdays	[–]	152	[61]
Fraction of woody parts of the total food	frw	[–]	0.268	[72]

The supply of available browse is calculated based on the assumption that only trees up to 5 m provide forage [73], but they are only browsed up to a height of 1.7 m, which is the maximum browsing height of red deer [74]. The quantity of available browse provided by a tree has been calculated as:



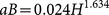



*aB*: available browse [kg dw]

*H*: tree height [m]

Browsing data for birch was derived from Kalén and Bergquist [73] and the reduced maximal foraging height for red deer was adopted. As no data was available for spruce, beech and oak, we also applied the biomasses estimated for birch to these tree species.

At each browsing event, a user-defined length of a branch (here set to 0.1 m) is eaten. The browsed part of the tree is converted to biomass following the allometric function [68]:



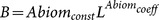



B: biomass [kg dw]

L: length of browsed branch [m]

Abiom_const_: allometric constant to calculate biomass [–]

Abiom_coeff_: allometric coefficient to calculate biomass [–]

Values for the two parameters, allometric constant (Abiom_const_) and allometric coefficient (Abiom_coeff_), were obtained by fitting this formula to our data on the twigs from collected trees on the Dreiborner Hochfläche (for details see [34]; raw data is publicly available online).

In the model, the ungulates are distributed evenly over the cells, in a way that in all cells the same amount of woody browse is eaten, if available. The browsed biomass from all trees and the surplus of germinating seedlings (Section 2.3.1) are summed up until the demand per cell is reached, thereafter browsing in this cell is stopped. For browsed trees, height growth is set to zero, and at the end of the year trees may die according to a species-specific browsing-induced mortality probability (Table 3). However, in the model, trees are only affected in terms of height growth by browsing up to a height of 1.7 m, when their leader shoot is still in reach of the maximum browsing height of red deer (even though trees up to a height of 5 m provide forage). Trees smaller than 0.2 m immediately die when being browsed. In the model, we did not consider any population dynamics of red deer, thus, the population density and the demand are kept constant during the simulation run. This can also be expected in reality due to actual hunting-related practices. In order to take the feeding behavior of red deer in the Eifel National Park into account, the individual trees in each cell are selected for browsing in the order of oak (most preferred), birch, beech, spruce (least preferred). Common broom does not appear to be browsed selectively in the Eifel National Park; therefore no browsing of broom is included in the model.

As thorny bushes such as blackthorn and bramble are known to act as nursery shrubs and to protect palatable tree species from browsing [56], [75]–[78], the demand of red deer is reduced in cells containing blackthorn and bramble by a factor F (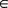
[0, 1]) according to a sigmoid function of cover:



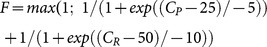



*F*: factor [–]

*C_P/R_*: cover of *P. spinosa* or *Rubus* sp. [m^2^]

Blackthorn was found to provide a higher protection from browsing in our study area than bramble [59], as is also calculated using the above equation.

## Results and Discussion

### 3.1. Model parameterization

Many of the model parameter values were obtained from the TreeMig and LandClim models. Remaining data gaps were closed by fitting parameter values to data collected for this study [34]. An overview of the parameter values is provided in Table 3.

#### 3.1.1. Growth of woody species

**Figure 3:**
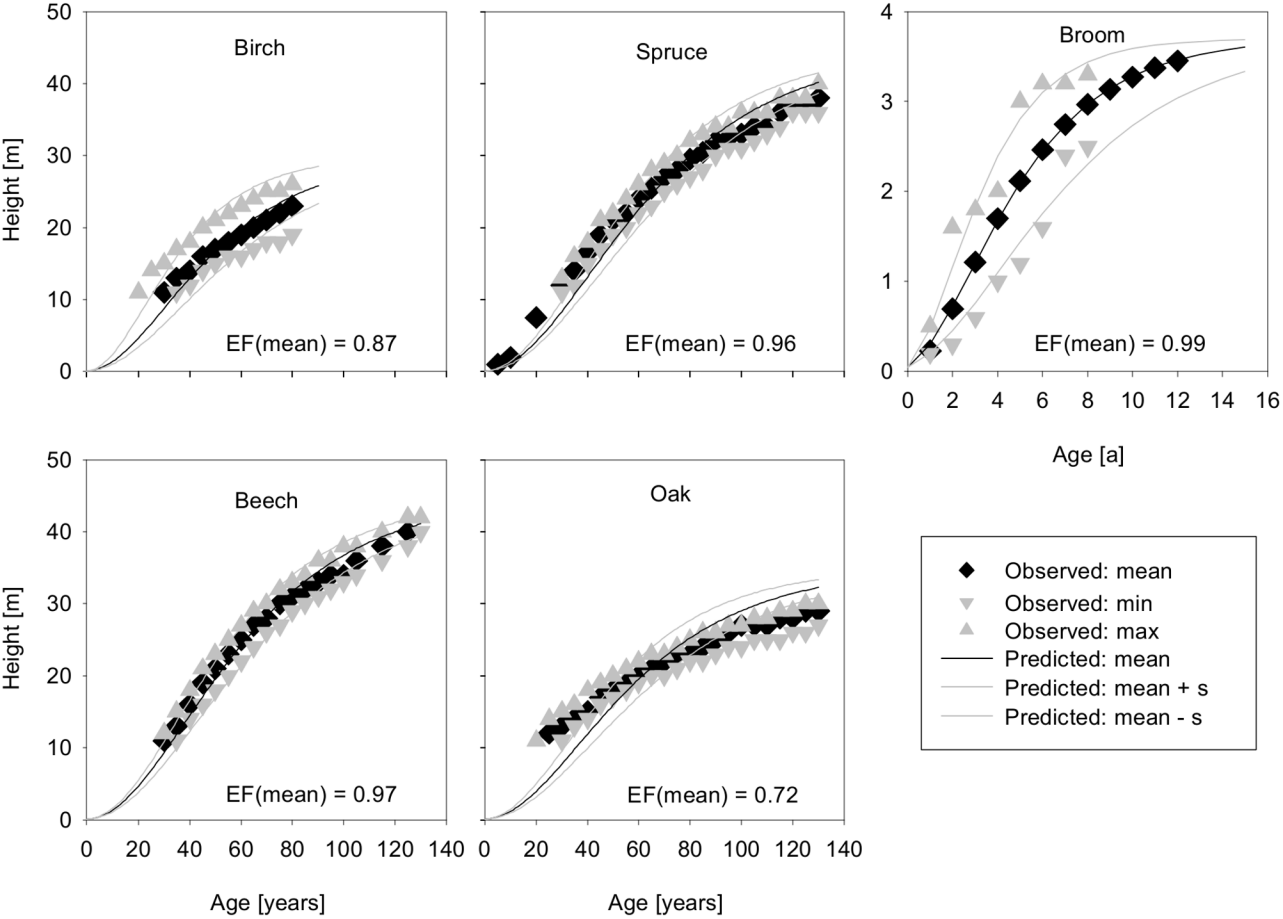
Simulated growth model (black line) fitting observed data of four tree species and one bush species. Model efficiency EF is calculated as described by [119]. Grey lines are the standard deviation (2s for broom).

Based on our observations in the Eifel National Park, initial values for the height of woody species were set to 0.05 m, which was the minimum height found for seedlings of the modelled species. The subsequent growth of trees considered in the model is shown in Fig. 3. In general, the model fits the growth data well as indicated by the model efficiencies. However, there was a lack of data on the height growth of young birch, beech and oak trees. In particular, for oak we observed a considerable deviation between model fit and data (Fig. 3).

Survival in young age is a bottleneck for wood establishment, often determining the course of succession. Small bushes and trees particularly suffer from competition and damage by browsing. An imprecise fit of tree growth might therefore lead to an over- or underestimation of establishment under browsing. More data on the growth and survival of young saplings and different species is needed to better understand the course of succession.

**Figure 4:**
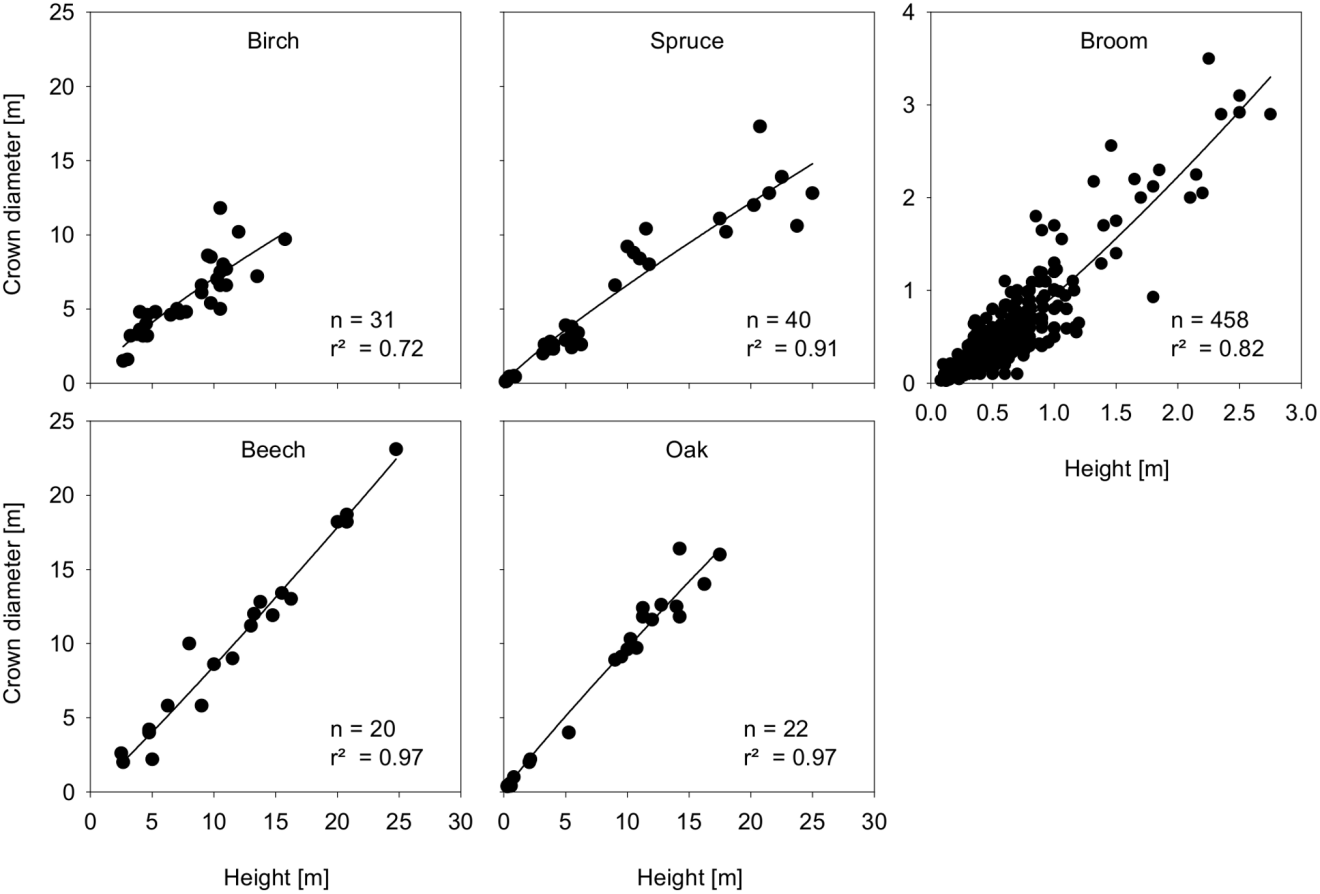
Crown diameter as an allometric function of height fitting observed data of solitary bushes and trees in the Eifel National Park. Raw data is publicly available online [34].

Data on crown diameters corresponding to the height of woody species are generally well represented by the model (Fig. 4).

#### 3.1.2. Seed dispersal and inhibition of wood encroachment by grasses

**Figure 5:**
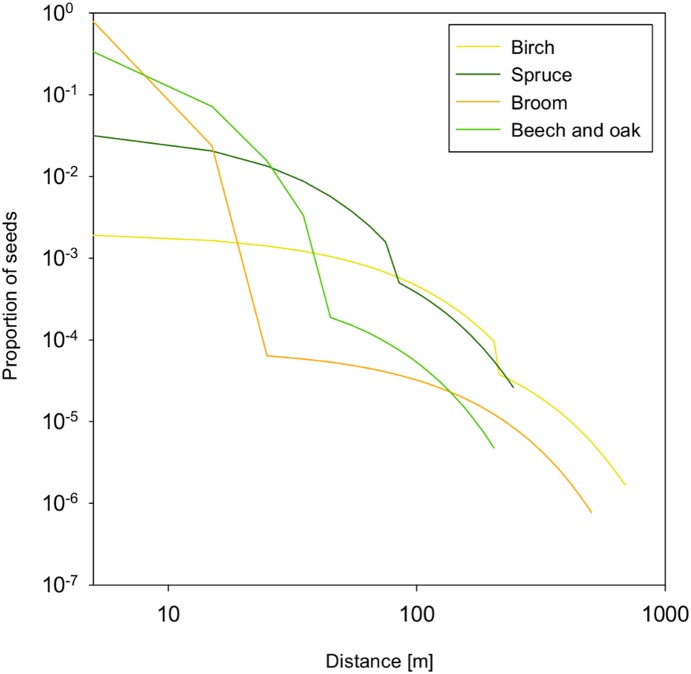
Proportion of seeds landing in the sink cell along one axis for studied bushes and trees starting from one parent plant as calculated from the LANDIS II double exponential seed dispersal function [49].

In the WoodS-Model we considered wind and zoochorous seed dispersal as illustrated in Fig. 5. The wind-dispersed species, birch and spruce, show a shallow dispersal curve with only a slight bend at the effective seeding distance. Broom, beech and oak, in contrast, disperse the biggest proportion of their seeds in the immediate neighborhood of the parent. Only a small number of seeds are distributed zoochorously over greater distances resulting in a sharp bend of the curves at the effective seeding distance (Fig. 5).

**Figure 6:**
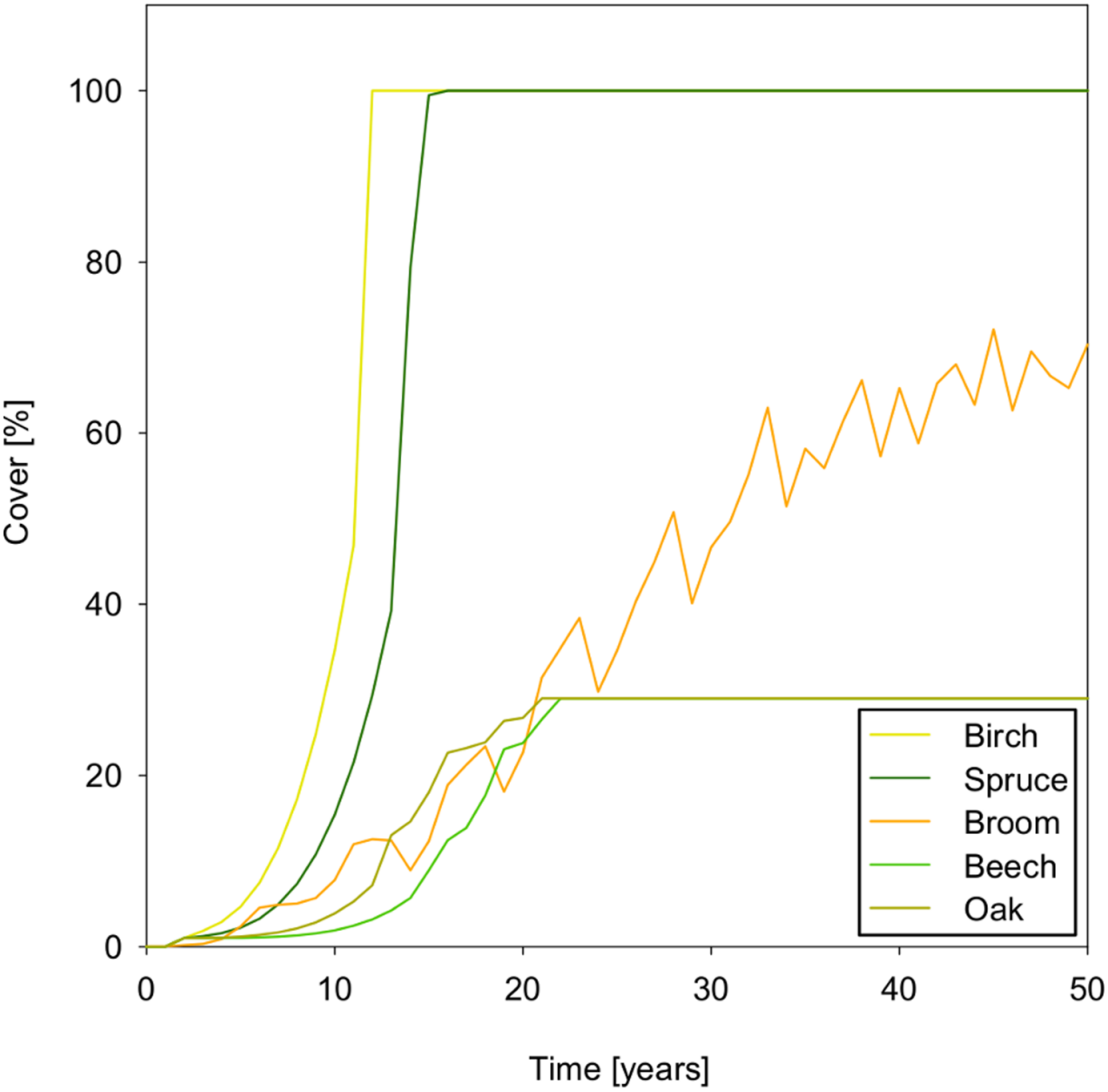
Wood encroachment starting with one parent plant in a 1 ha scenario (c.f. Fig. 7) without inhibition by the herbaceous layer.

The encroachment of broom and trees that emerges from seed dispersal, germination and growth, without any further calibration, is illustrated in Fig. 6 for the area of 1 ha ( = 10 cells×10 cells) starting with one parent plant. The wind-dispersed species, birch and spruce, spread fast and cover the simulated area in 12 and 16 years, respectively. Beech and oak spread more slowly, both reaching a maximum cover of 29% in 22 years.

**Figure 7:**
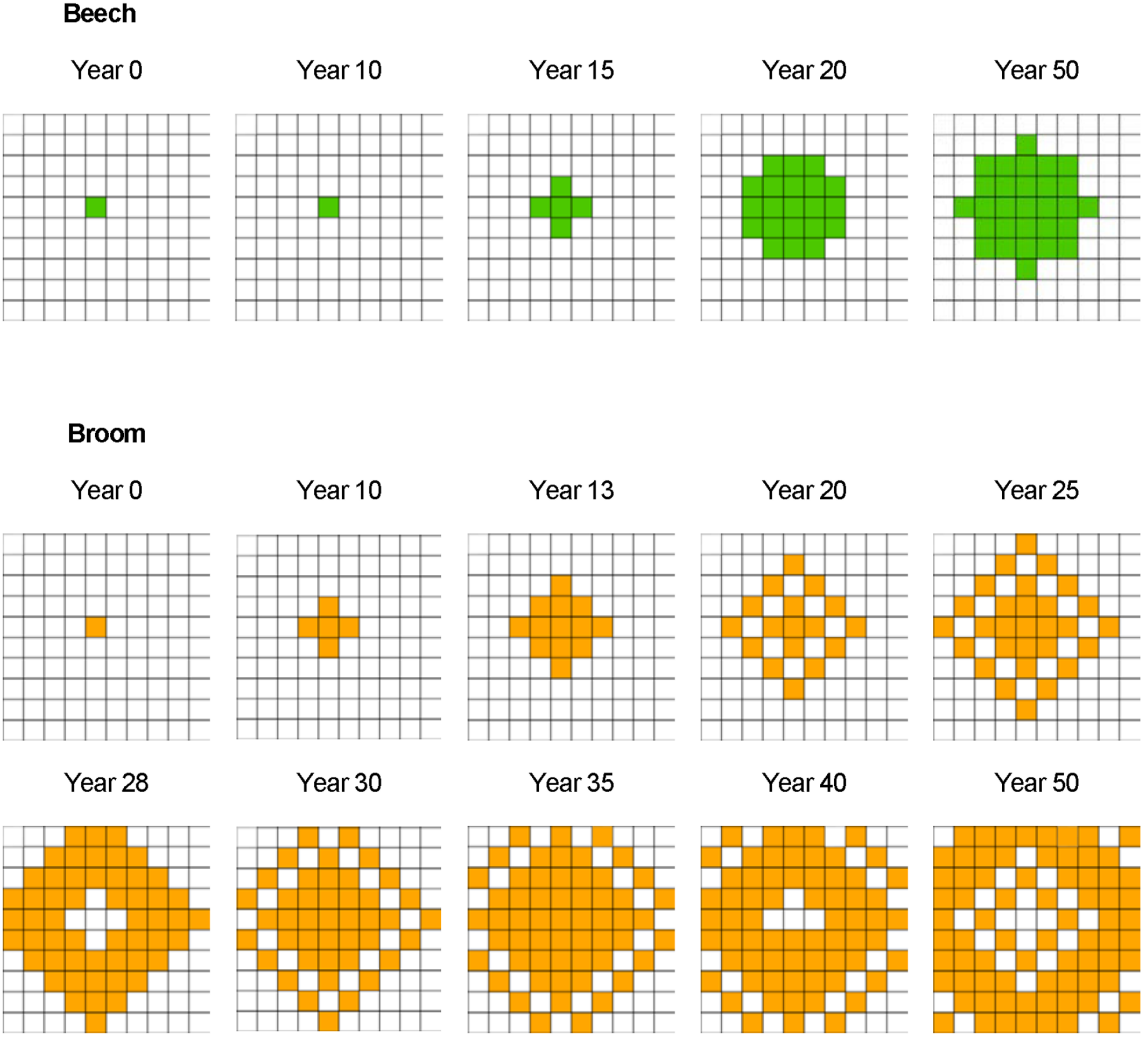
Encroachment of beech (green: cells with a cover >50%) and broom (orange: cells with a cover >50%) on an area of 1 ha starting with one parent plant without inhibition by the herbaceous layer and not influenced by red deer.

Spatial spread of beech is illustrated in Fig. 7 for the same simulation run as shown in Fig. 6. In the model, the spread of beech stops when all cells within the effective seeding distance of 30 m are covered by beech. Zoochorous dispersal does not take place in this scenario, as the lack of bushes or woods would not attract jays. A further spread from the newly conquered cells does not take place within the simulated time frame, because it takes a beech tree 50 years to reach maturity. Broom shows oscillating population dynamics over time (Fig. 6) and space (Fig. 7). This oscillation might be the result of the interplay between fast maturation and death of broom individuals: fast maturation, just four years after germination, can lead to a rapid increase in population size, while the death of broom individuals at the low maximum age of 12 years leads to a decline [79], [80].

Germination of dispersed seeds, and also wood establishment, might be hindered by dense grass swards, as grasses compete with young saplings for light, nutrients and water [55], [81]–[83] and might suppress the germination and establishment through allelopathic effects [55], [84]. To account for these mechanisms but at the same time reduce complexity, these different processes were lumped into a single inhibition function. The inhibition factor has been calibrated using observed patterns as follows. We started the calibration process based on broom, because most data was available for this species. Therefore, several pastures and meadows that had recently been abandoned were investigated by counting the emerging young broom individuals in the center of the pastures and calculating the mean abundance per 100 m^2^, i.e. the size of one cell (Table 5). When investigating meadows, we made a distinction between the abundance of broom seedlings close to the shrub edge and the abundance in the center of the meadow. At the edge of meadows adjoining broom, i.e. within the reach of ballistic seed dispersal, many seedlings emerged in the first year after abandonment before a thick grass felt developed (Table 5). In the center of the abandoned meadows that were surrounded by broom, a few solitary bushes were established within the first year after abandonment, possibly as a result of zoochorous seed dispersal [85], [86]. In the center of the abandoned pastures, a higher number of broom bushes was observed to become established during the first year after abandonment (Table 5). The investigated fallows were surrounded by differing numbers of broom bushes. For the calibration we thus created a standard scenario using the mean surrounding broom abundance. During the calibration process, broom individuals per cell were counted, and the mean number of individuals per cell was compared to observed data (Table 5).

**Table 5:** Observed mean number of broom individuals (Ind) in recently abandoned meadows and pastures on the Dreiborner Hochfläche (Heilburg (2008, unpublished), Krämer [58], Engler [57] and own data), and simulation results after calibration. n: number of field surveys.

	Meadow (edge)	Meadow (middle)	Pasture (middle)
Observation [Ind 100 m^−2^] (n)	6.5 (6)	0.2 (10)	5.2 (16)
Simulation results [Ind 100 m^−2^]	8.3	0.2	5.2

At the Dreiborner Hochfläche, broom was unable to reproduce in fallow grasslands that had developed thick grass felts [58], [62]. A similar inhibition was also observed for other sites [67], [80], [87], [88]. To account for this observation, the parameters I_50_, p(ED) and S (Table 3) were calibrated in a way that the simulated mean number of broom individuals per cell recruited in the first year met the observed data (Table 5) and no further broom recruitment took place in the following simulation years.

**Figure 8:**
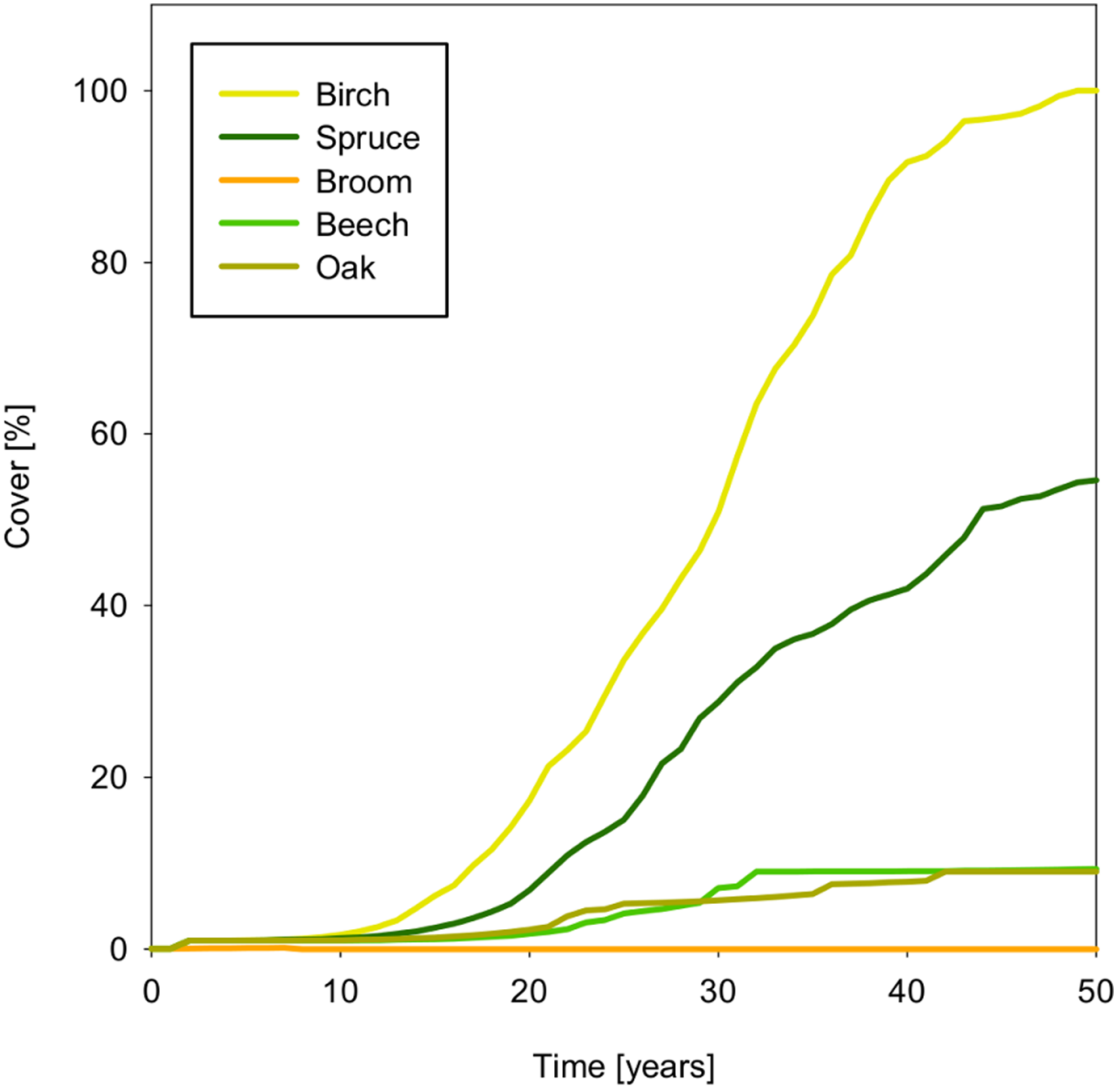
Wood encroachment on a fallow grassland starting with one parent plant in a 1 ha scenario not influenced by red deer but considering the inhibition of the herbaceous layer.

Wood encroachment in abandoned grasslands at the Dreiborner Hochfläche is slow [61], [62]. To account for this delay in our model, we considered the inhibition of the input into the seed bank and subsequent germination of seeds by the herbaceous layer. To calibrate the inhibition function, we increased the parameter value until a delayed wood encroachment took place in a fallow of 1 ha ( = 10 cells×10 cells), starting with one mature tree or bush and a value for the parameter I_50_ as estimated for broom (see above). The resulting simulations are displayed in Fig. 8.

#### 3.1.3. Ungulate browsing

**Figure 9:**
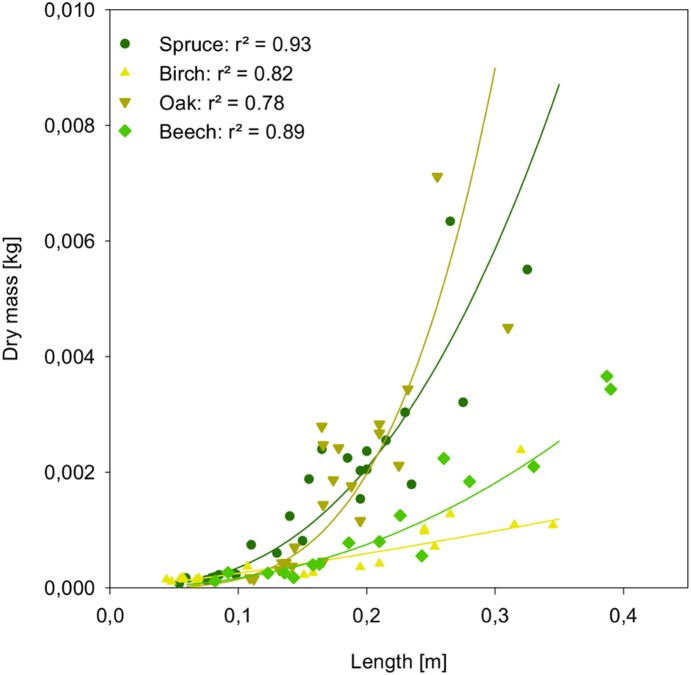
Biomass of twigs as an allometric function of length fitting observed data of collected twigs (raw data is publicly available online [34]).

We explicitly modelled the influence of ungulate browsing on tree establishment under consideration of the actual animal density in a given area. Therefore, at each simulated browsing event, a defined length of branch is eaten, and the length of branches is subsequently converted into biomass as shown in Fig. 9.

For reasons of simplicity we did not consider seasonal or site-specific variation in feeding rates or food preference. However, the portion of woody browse in food and feeding preferences within woody browse might vary strongly among different sites [89], [90]. Some more detailed information on the portion of woody browse in the diet of red deer is available from [72], [89], [91]. Red deer feed mostly on coniferous browse during the winter [92], when not much other food is available, whereas deciduous trees are more often browsed in summer [89]. Such peaks in browsing intensity during a certain time of the year might lead to greater damage than a uniform distribution, especially because trees might react differently to summer and winter browsing [93]. Furthermore, the frequency of browsing over the year might affect the browsing-related mortality [94], where smaller trees might suffer from a higher mortality than taller ones [95]. More browsing studies with an experimental design such as the ones by Harmer [96] and Vandenberghe et al. [95] are needed in order to understand the impact of ungulate browsing on tree regeneration and subsequently wood encroachment and to gain a database to parameterize browsing models for different tree species.

A further simplification in our model is that ungulate density remains constant over space and time. Yet browsing intensity is often clumped in space [89] and animal density might vary over time. Earlier simulation studies showed that this variation might influence forest regeneration, creating windows of opportunities for trees to become established [97]–[99]. The aforementioned data gaps and oversimplifications are likely to affect our equation comparing the supply and demand of woody browse. Yet, integrating all these additional processes and factors would lead to a more detailed model that might ensure more sophisticated simulations but, in turn, would hamper the use of the model on a large area and make parameterization even more difficult. Simulating browsing effects on a finer scale would require a much more detailed model including microclimatic conditions as done for the HUNGER model [20], which operates based on patch sizes of, for example, 0.001 ha.

### 3.2. Pattern-oriented model test in a landscape context

One of the main assumptions in the theory of succession is that the initial vegetation state and neighborhood relationships strongly influence the course of succession, which is also the reason why succession often strongly varies among sites [11]–[15]. In order to verify this assumption and additionally evaluate the effect of browsing intensity, we tested the model performance in the landscape context of our study site. First, we compared the simulation results of the status quo scenario of 22 red deer per 100 ha, which is one of the highest population densities in Central Europe [100], [101], to observed landscape data [34]. Second, we investigated successional trajectories and corresponding landscape patterns employing different abundances of red deer. The simulated landscapes are independent secondary predictions and were not taken into consideration during model development and parameterization, i.e. they were not predetermined by any input parameters. The pattern-oriented model test thus provides strong evidence of the predictive capability of the model [102].

#### 3.2.1. Comparison of observed field data and simulation results

**Figure 10:**
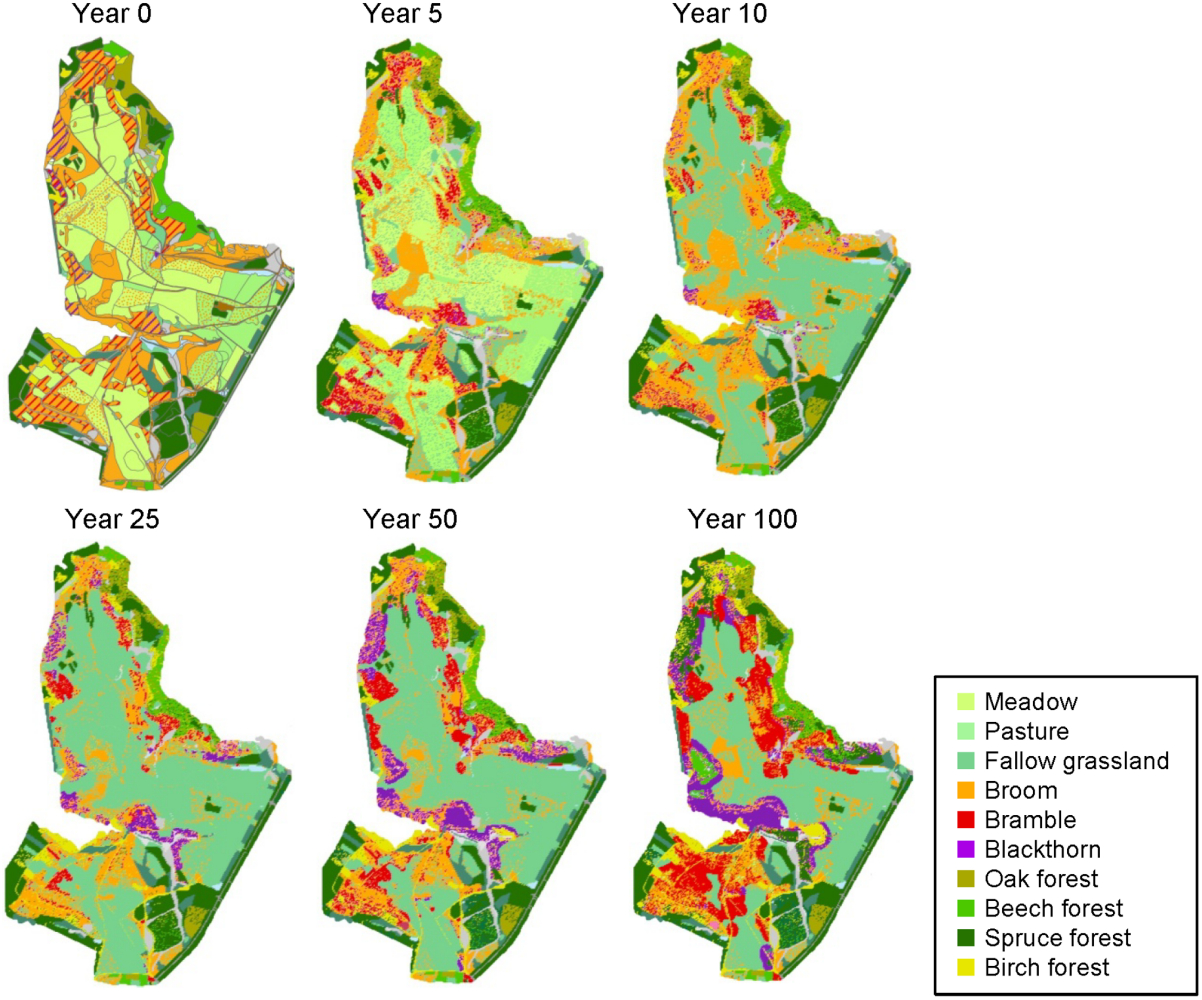
Simulation of the landscape development in the southern part of the Dreiborner Hochfläche with the actual population density of red deer (22 animals per 100 ha). Names of bushes refer to shrub patches with a bush cover ≥10%. In forest cells, cover of trees is ≥50%.

In order to provide independent data for a plausibility check of model performance, successional patterns were mapped in several field surveys of fallow areas at the Dreiborner Hochfläche [34], [57], [59], [69], for which the time of abandonment could be reconstructed from aerial photographs [59]. In order to compare the observed patterns with the model output, a part of the Dreiborner Hochfläche was simulated under conditions matching those in the areas under investigation, i.e. abandoned areas with the status quo density of red deer. The detailed vegetation map of the Dreiborner Hochfläche [33] used to initialize simulations (year 0) and the model predictions for subsequent years are shown in Fig. 10.

**Figure 11:**
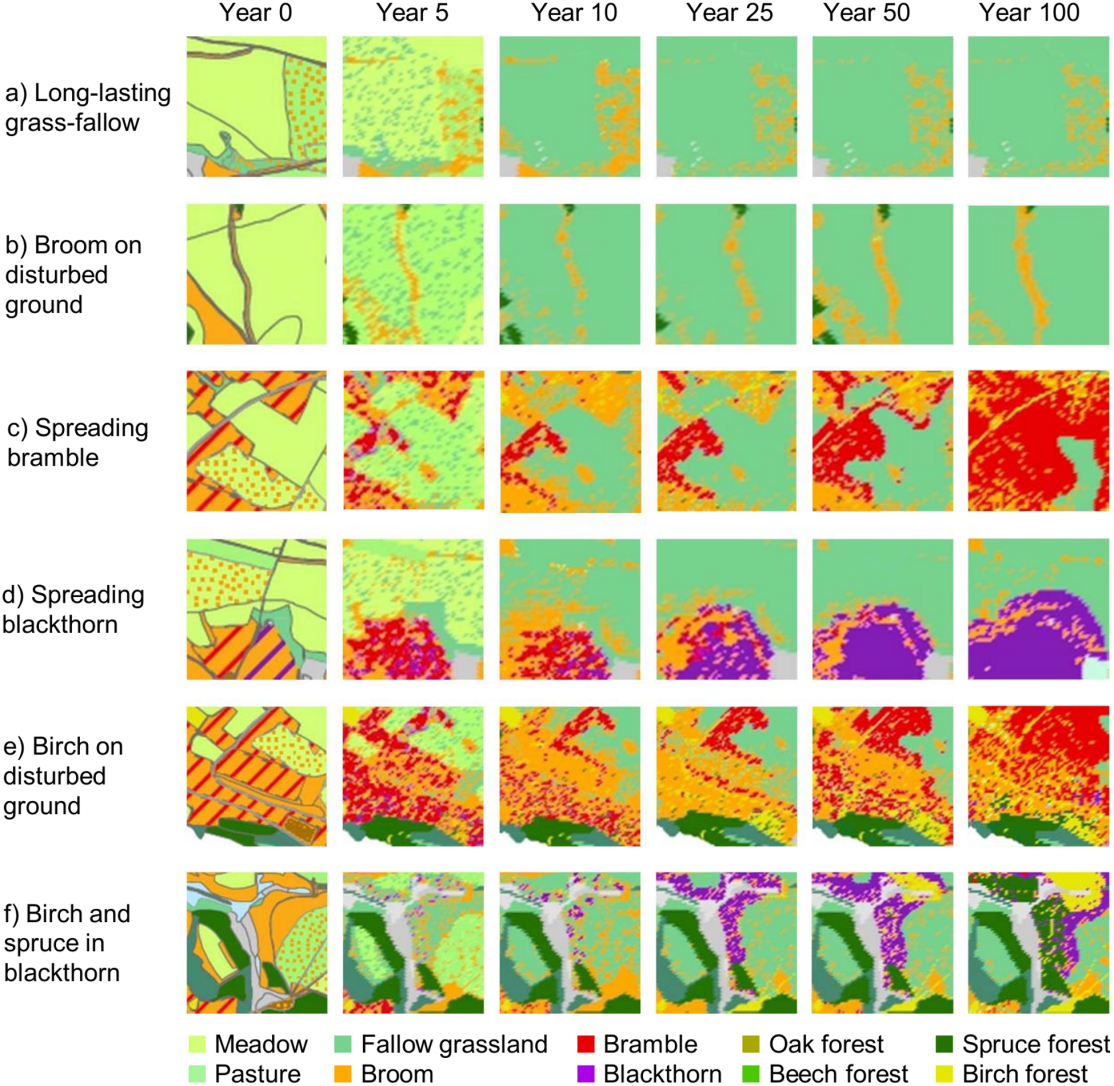
Simulation: Vegetation dynamics depending on initial state and neighborhood. Details of the simulation shown in Fig. 10.

The simulated vegetation succession strongly depended on neighborhood relationships and therefore on initial vegetation as shown for partial maps in Fig. 11.

**Figure 12:**
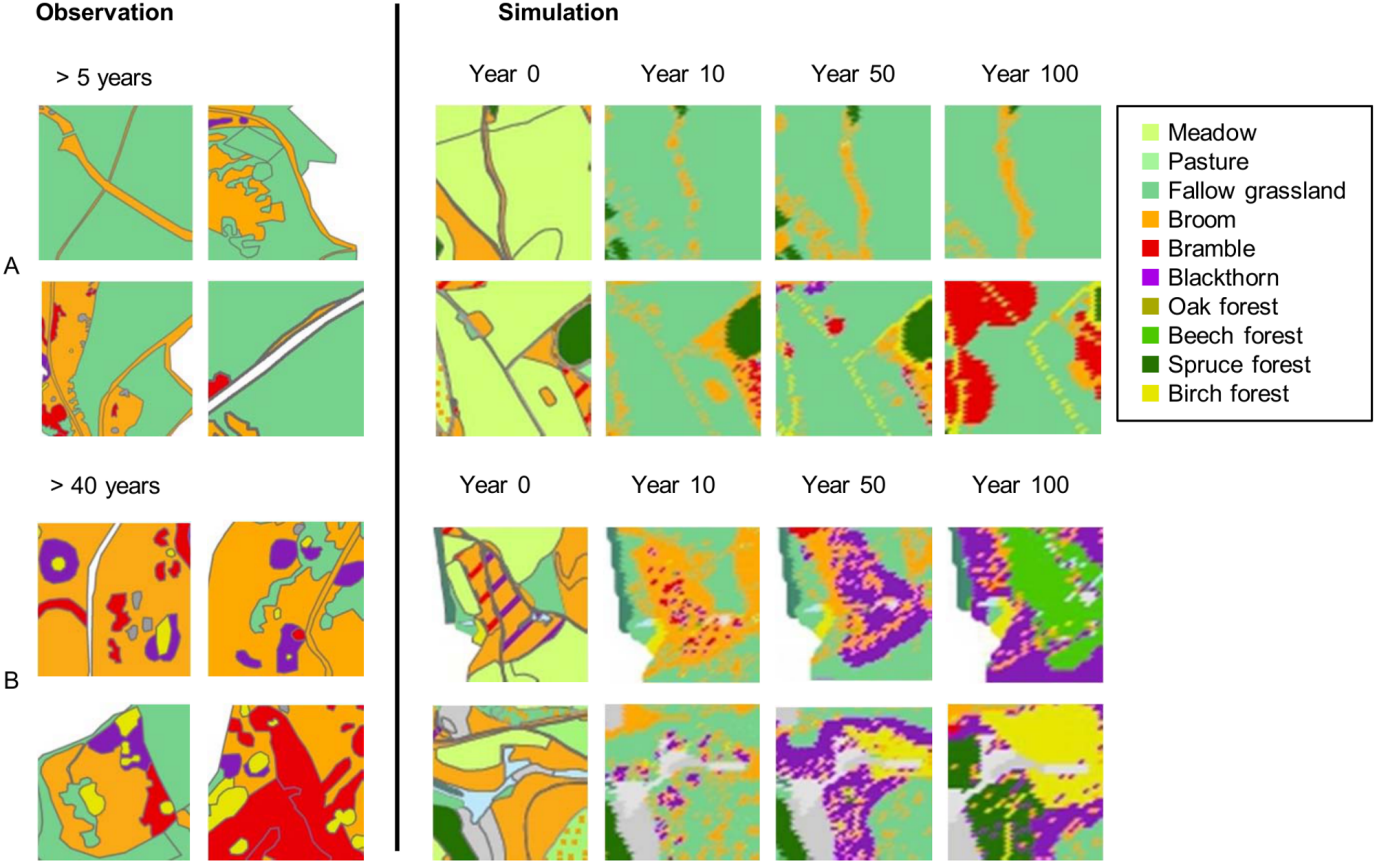
Observation vs. simulation: Examples of typical patterns of succession from vegetation mapping [59] of areas of known age after abandonment, and results of simulations showing same successional patterns over time. A: Encroachment of broom on abandoned field paths. B: Spread of bramble and blackthorn into fallow grasslands and subsequent development of new forests from within thorny scrub. Since the observation is time point 0 for the simulation, a direct comparison is not possible. Therefore, we show examples of typical patterns in the observation and simulation for the same landscape. The intention is not to provide a direct comparison, but the changes of landscape structure within a given time frame.

Grassland areas decreased over time, as they were overgrown by bramble and blackthorn, where initials of these bushes were present (Fig. 10, Fig. 11c and d, Fig. 12 b). Yet, wide grassland areas in the center of the sites remained unwooded even after a simulation time of 100 years (Fig. 10, Fig. 11a). In simulations, fallow grassland was observed for some sites that had been abandoned for more than 40 years even close to neighboring forests (Fig. 10). The dynamics of the herbaceous layer influenced the succession of woody species, because the development of fallows dominated by tall grasses led to a strong inhibition of wood encroachment.

Broom showed oscillating population dynamics with a slight decreasing trend in the simulation (Fig. 10). Broom did not grow in fallow grassland areas, but established itself on disturbed ground, such as former field paths and sites of military disturbance (Fig. 10, Fig. 11b), and was observed on fallow field paths five years after abandonment (Fig. 12a). Field monitoring on the Dreiborner Hochfläche revealed broom establishment even though there were no propagule sources nearby [59], probably as a result of germination from long-lasting seed banks observed for this species [103], [104]. In the model, seed banks that established themselves before the start of simulation were not integrated, because their existence could not be reconstructed in the required detail. Consequently, the recruitment of broom might be underestimated in our simulation.

Bramble and blackthorn were found to play a major role in the succession on the Dreiborner Hochfläche, providing sites that were excluded from browsing (see Figs. 10–12). As a result, patches of young forest islands, surrounded by a ring of thorny bushes, were formed within 40 years of abandonment in both field observations and model simulation (Fig. 12b).

**Figure 13:**
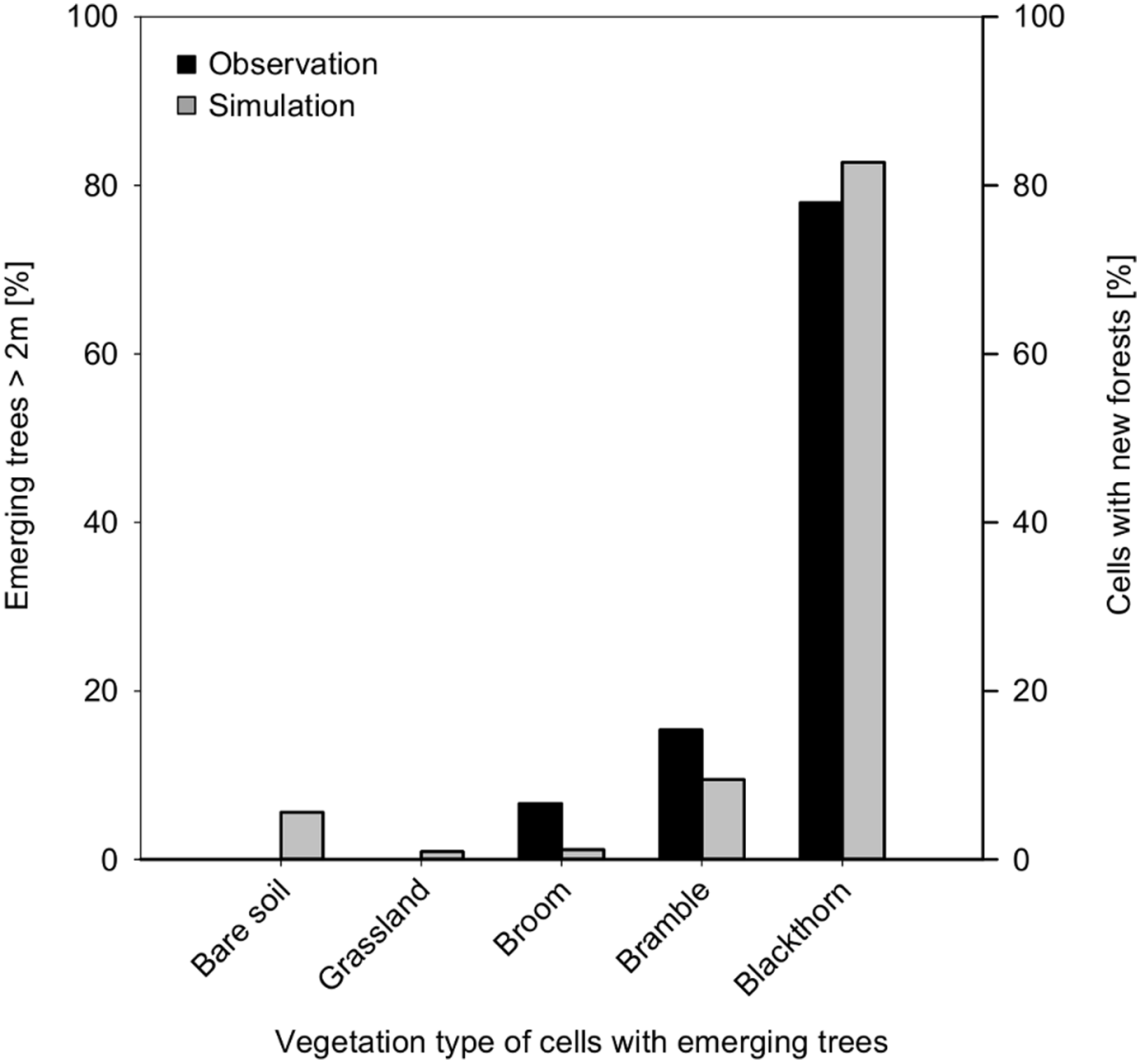
Observation vs. simulation: Black bars are related to the relative number of established trees observed in the field surveys with a height >2 m [34], [57], [59], [69]. Grey bars are related to the number of simulated cells with emerging new forests.

In simulations as well as in field observations, the establishment of broom and trees was strongly hindered by dense populations of tall grasses. Regarding the association of saplings with nurse plants, simulation results align with field observations: saplings with a height > 2 m can be thought to have escaped severe browsing damage of the leader shoot, so that wood encroachment is more likely to take place. Most (78%) of the young trees of this height have been observed in blackthorn thickets, some (15%) in bramble, a few (7%) in broom thickets, and none on grassland and disturbed ground (Fig. 13), compared to simulated values of 83% for blackthorn thickets, 9% for bramble and 1% for grassland and broom each. A major difference between simulation and observation is that 6% of simulated cells, which were initially covered by bare soil, turned into new forest cells, whereas in the field no trees >2 m were observed on these formerly disturbed grounds. This difference might be due to the way ungulate browsing is implemented in the model. In our approach, we integrated the interaction of general tree recruitment capability and browsing effect, which is in turn influenced by the availability of woody browse. As a consequence, lower numbers of saplings result in higher browsing effects by a given density of ungulates. In contrast, at higher numbers of emerging seedlings, a given density of ungulates will not necessarily hinder the establishment of all saplings, an effect that has already been discussed by Augustine and McNaughton [99]. However, in our model, ungulate browsers are evenly distributed over the simulated cells. This may lead to an underestimation of browsing in particularly attractive cells. The importance of open ground along field paths as suitable sites for wood establishment may therefore be even higher in reality, as is already shown in the simulation. In the case of disturbed ground occurring in the vicinity of birch, simulated seedling establishment was high enough to fulfil the demand of red deer so that a sufficient number of trees survived to form a young wood patch. For the Eifel National Park it is also important that disturbance of the ungulates by visitors along frequently used field paths is not integrated in the model and browsing along these areas may therefore be overestimated in our simulation.

The development of simulated new forest cells often took 35 or even 50 to 100 years (Fig. 10, Fig. 11f, Fig. 12b). This time frame for the establishment of new forest cells is in accordance with field observations: the establishment of trees in thorny bushes was observed on sites that had been abandoned for more than 40 years (Fig. 12b). An exception to this time frame was observed for simulated birch trees becoming established on previously disturbed sites. Considerable birch recruitment took place after just 25 years on certain grassland patches that had become established on disturbed ground, such as field paths (Fig. 10, Fig. 11e). This type of grassland vegetation was not dense and was dominated by tall grasses. The number of oak and beech forests did not increase in any of the simulations. Please also note that succession within forests (e.g. from birch to beech or oak) is not included in our model.

The simulation of succession on formerly used grasslands at the Dreiborner Hochfläche produced patterns that are well documented throughout the literature: grasslands and broom shrubs are slowly overgrown by shrubs [105], [106], which act as “nurse plants” [77], [107] enhancing wood encroachment. Where no initial shrubs or disturbed sites exist within the dispersal distance of adult trees, fallow grasslands persist over several decades [11], [13]. To our knowledge, the important role these thorny bushes play in the encroachment of wood has not been integrated into other landscape models. In general, little is known about the establishment niches of these bushes [78], [108], despite their importance for the transition of grassland into wooded landscapes [9], [77], [78], [108]–[110].

In general, our model test reveals that the WoodS-Model is able to emulate observed patterns with the resolution of 100 m^2^ raster cells, so that the loss of detail seems to be acceptable in the simulation on the landscape scale.

#### 3.2.2. Model behavior with different population densities of red deer

**Figure 14:**
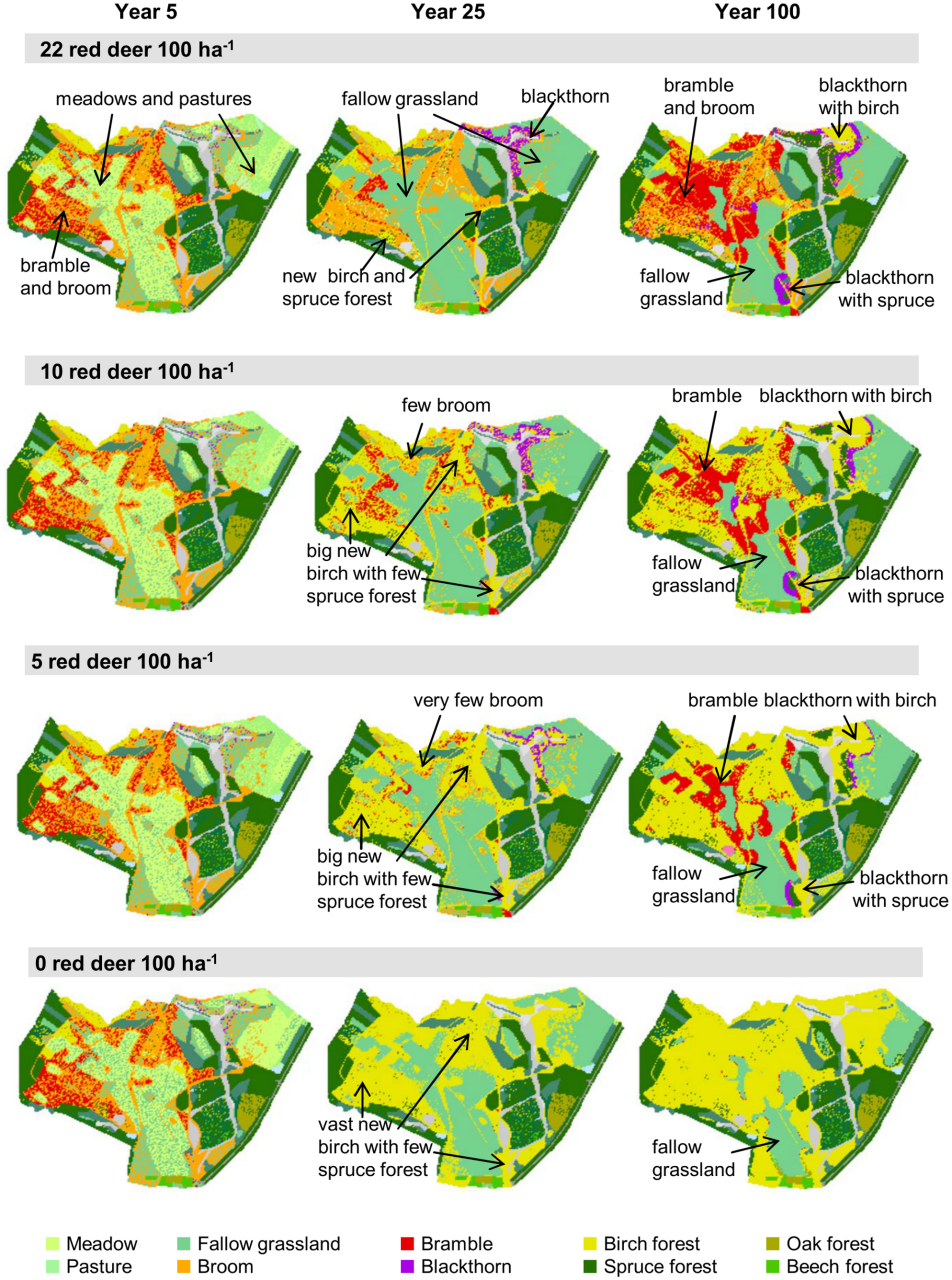
Simulated vegetation development of the southern part of the Dreiborner Hochfläche if abandoned with different population densities of red deer. Initial vegetation composition as in Fig. 10.

**Figure 15:**
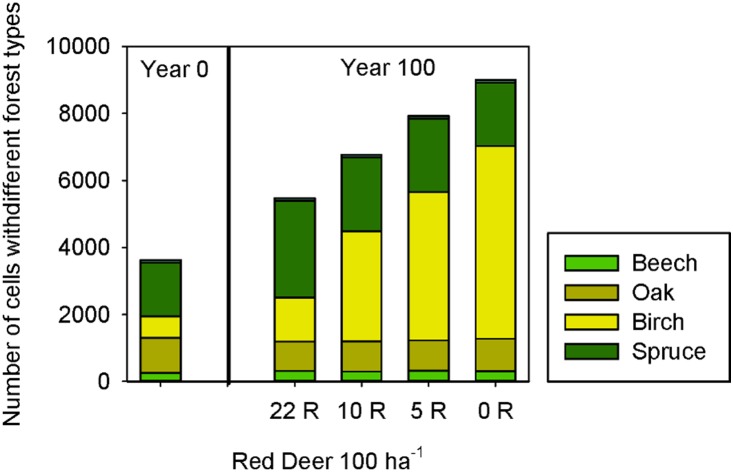
Forest development after 100 simulated years in the southern part of the Dreiborner Hochfläche if abandoned with different population densities of red deer (R). Same simulation as in Fig. 14. Please note that succession within forests (e.g. from birch to beech or oak) is not included in our model.

In order to test the model with different abundances of red deer, we focused on the southern part of the Dreiborner Hochfläche. To check the plausibility of the model behavior and evaluate the possibility of using the WoodS-Model for decision support in wildlife management, we compared model results as derived from the status quo density simulations (Section 3.2.1) with simulated vegetation developments for lower population densities of 0, 5 and 10 red deer per 100 ha^−1^ (Figs. 14 and 15).

The predicted landscape development following abandonment strongly depended on the density of red deer in the area (Figs. 14 and 15). When considering a high density of 22 red deer per 100 ha^−1^ in the simulation, a few wood patches developed first along abandoned field paths and later within blackthorn shrubs (Fig. 14). New emerging wood patches were formed by birch, and, to a very small extent, also by spruce. Thickets of bramble and blackthorn spread into former grasslands. While blackthorn was subsequently overgrown by emerging trees, starting at the center of the thickets, large bramble thickets persisted for more than 100 years. Broom was able to persist at sites where it was present initially, but in contrast to bramble and blackthorn did not spread into the fallow grasslands. Also, vast areas with fallow grasslands were developing a dense sward, which, in combination with the high browsing pressure from red deer, suppressed wood encroachment in these areas. The exceedingly high population density of red deer kept the succession towards forests almost in check. Usually, shrubs do not persist over such a long period of time, but are overgrown by trees [8], [105], [106], [111]–[115]. In this regard, it is therefore difficult to assess the further development and to evaluate model predictions. It is not clear whether, in reality, bramble and other shrub patches can persist and keep spreading over a period of 100 years as demonstrated in the simulation (where they do not age and die of senescence) or whether they degenerate and probably revert to fallow grasslands in case no tree encroachment takes place. Furthermore, bramble and blackthorn in our model only disperse vegetatively; dispersal of the berries by birds has not been accounted for. Therefore, the development of bramble and blackthorn thickets relies on initials of these bushes in the input layer, so that important “nurse” spots may be under-represented in the model.

At lower densities of 5 and 10 red deer per 100 ha^−1^, more birch forests developed compared to status quo density simulations, which were restricted mainly to former broom and bramble patches. However, the amount of emerging spruce remained more or less the same (Figs. 14 and 15). These new forests overgrew all broom shrubs and most of the bramble and especially blackthorn thickets, so that only small areas of these thorny bushes remained at the borders between forests and fallow grasslands. In line with many grassland succession trajectories [8], [105], [106], [111]–[116], in our simulations using the non-interference scenario without any red deer, existing shrub patches were rapidly overgrown by emerging trees, and most grassland areas developed into woods, mainly birch forests, over the simulation period of 100 years. Fallow grasslands that are located at a distance from seed sources remained unwooded due to low seed availability and high inhibition of emergence by grasses. Even though the forests kept encroaching on the fallow grasslands, big unwooded areas remained after 100 simulated years.

Overall, the model produces plausible simulation results with different abundances of red deer. The simulated restricted wood encroachment with the current extraordinary high population density is in line with the observations, whereas regularly observed succession [105], [112], [113] takes place under lower browsing pressure.

## Conclusion

The comparison of landscape development patterns emerging from the simulated processes with observed data reveals that the WoodS-Model is able to adequately mimic spatio-temporal dynamics on the landscape scale. Based on the principle of structural realism [117], observed patterns have been reproduced in a highly realistic way on a spatial scale for the example of shrub development, and on a temporal scale for short- to middle-term succession patterns. We showed that the interacting processes of competition for space, wood recruitment, inhibition by the herbaceous layer, ungulate browsing and facilitation by nurse plants are sufficient to emulate observed patterns, whereby model parameterization is based, to a large extent, on field data. The model thereby provides a highly detailed picture of succession at the given landscape by considering neighborhood relationships in the spatio-temporal frame of grassland-to-wood succession.

Vegetation development varies among different parts of the landscape. Small structural landscape elements such as abandoned field paths, or initial shrub and wood patches, are capable of considerably determining vegetation development. In particular, the location of seed sources such as mature bushes and trees influences wood encroachment, and once a seed has reached a particular site, local patch conditions need to allow for a successful establishment of young saplings. Moreover, biotic interactions, such as germination depending on herbaceous dynamics and ungulate browsing, can be crucial for the course of vegetation development. Structural landscape elements and initial vegetation composition are known to determine the course of succession, in particular for wood encroachment on grasslands, so that predictions are often difficult to make [15], [118]. Neighborhood effects, which may determine, for instance, the existence and location of seed sources and initials of shrubs, have been shown to highly influence the succession in both our simulation study and our space-for-time field observations.

Process-based and spatially explicit modeling approaches, such as the presented WoodS-model, allow neighboring and biotic interaction in the evaluation of certain isolated processes in various landscape scenarios to be accounted for, and predictions about future landscape states to be made. The advantage of the WoodS-Model as a decision support system in particular is that it integrates many important and mutually interacting processes determining the course of succession. Due to the generality of these processes, the model can easily be transferred to other sites and, thus, has a potentially widespread applicability. The assessment of projected impact on the landscape as a result of different simulated management scenarios can assist stakeholders in choosing suitable strategies regarding open grassland management.
